# Calprotectin-mediated survival of *Staphylococcus aureus* in coculture with *Pseudomonas aeruginosa* occurs without nutrient metal sequestration

**DOI:** 10.1128/mbio.03846-24

**Published:** 2025-03-28

**Authors:** Wei H. Lee, Emily M. Zygiel, Celis H. Lee, Amanda G. Oglesby, Elizabeth M. Nolan

**Affiliations:** 1 Department of Chemistry, Massachusetts Institute of Technology 2167 , Cambridge, Massachusetts, USA; 2 Department of Pharmaceutical Sciences, School of Pharmacy, University of Maryland, Baltimore, Maryland, USA; 3 Department of Microbiology and Immunology, School of Medicine, University of Maryland, Baltimore, Maryland, USA; Instituto de Biologia Molecular y Celular de Rosario, Rosario, Santa Fe, Argentina

**Keywords:** *Pseudomonas aeruginosa*, *Staphylococcus aureus*, calprotectin, iron, nutritional immunity

## Abstract

*Pseudomonas aeruginosa* and *Staphylococcus aureus* are bacterial pathogens of major clinical concern that cause polymicrobial infections in diverse patient populations. Human calprotectin (CP; S100A8/S100A9 heterooligomer, MRP8/MRP14 heterooligomer) is a host-defense protein that contributes to nutritional immunity by sequestering multiple nutrient metal ions including Mn(II), Fe(II), and Zn(II). Here, we examine the consequences of metal availability and CP treatment on cocultures of *P. aeruginosa* and *S. aureus*. We report that CP elicits Fe-starvation responses in both *P. aeruginosa* and *S. aureus* in coculture, including the upregulation of genes involved in Fe uptake by both organisms. Moreover, analyses of pseudomonal metabolites in coculture supernatants further demonstrate Fe-starvation responses, showing that CP treatment leads to increased siderophore levels and reduced phenazine levels. Consistent with prior studies, growth under conditions of Fe depletion accelerated *P. aeruginosa* killing of *S. aureus* in coculture, but treatment with CP promoted *S. aureus* survival. Treatment with CP site variants lacking functional transition-metal-binding sites and metalated CP also enhanced *S. aureus* survival in coculture with *P. aeruginosa*, revealing that this consequence of CP treatment is independent of its canonical metal-sequestering function. Thus, the protective effects of CP treatment during coculture appear to override the observed Fe-starvation effects that make *P. aeruginosa* more virulent toward *S. aureus*. This work highlights an unappreciated facet of how CP contributes to host–pathogen and pathogen–pathogen interactions that are relevant to human infectious disease.

**IMPORTANCE:**

The current working model that describes how the innate immune protein calprotectin (CP) protects the host against bacterial pathogens focuses on its capacity to sequester multiple essential metal nutrients in a process called nutritional immunity. Our study further explores this function by focusing on the effects of metal availability and CP treatment on the dynamics of *Pseudomonas aeruginosa* and *Staphylococcus aureus* grown in coculture. These two bacterial pathogens are of significant clinical concern and colocalize with CP at infection sites. This work reveals that CP modulates *P. aeruginosa/S. aureus* coculture dynamics in a manner that is independent of its ability to sequester nutrient metal ions. This surprising result is important because it demonstrates that CP has metal-independent function and thus contributes to the host–pathogen and pathogen–pathogen interactions in ways that are not accounted for in the current working model focused on metal sequestration.

## INTRODUCTION

*Pseudomonas aeruginosa* and *Staphylococcus aureus* are leading causes of life-threatening, antimicrobial-resistant, polymicrobial infections in diverse patient populations (1–3). These include surgical wound infections, infected diabetic foot ulcers, and chronic pulmonary infections in individuals with cystic fibrosis (CF). The clinical relevance of *P. aeruginosa*/*S. aureus* communities has motivated fundamental studies of *P. aeruginosa*/*S. aureus* coculture dynamics, including work that revealed molecular factors that mediate these interspecies interactions and how they are impacted by host immunity (4–6).

A hallmark of *P. aeruginosa*/*S. aureus* cocultures is that *P. aeruginosa* exerts antimicrobial activity toward *S. aureus* through the action of multiple molecular factors. The quorum sensing molecule 2-heptyl-3-hydroxy-4-quinolone (PQS) produced by *P. aeruginosa* leads to the upregulation of many anti-staphylococcal factors (7, 8). Among these are the staphylolytic protease LasA and small molecules that include redox-cycling phenazines, hydrogen cyanide, and 2-heptyl-4-hydroxyquinoline 1-oxide (HQNO) (9–14). Each of these small molecules produced by *P. aeruginosa* inhibits *S. aureus* respiration, which causes *S. aureus* to shift toward fermentative metabolism, resulting in reduced *S. aureus* growth rates and the subsequent emergence of small colony variants that are antimicrobial-tolerant (10, 13, 15). The *P. aeruginosa* siderophores pyoverdine (PVD) and pyochelin (PCH) that scavenge Fe(III) are also required for *P. aeruginosa* to cause *S. aureus* to shift toward fermentative metabolism (15–17). Moreover, Fe starvation was shown to enhance the production of PQS and HQNO by *P. aeruginosa*, leading to enhanced antimicrobial activity against *S. aureus* (18, 19). Furthermore, two recent reports detected methylated PCH in *P. aeruginosa*/*S. aureus* cocultures and identified a gene encoding a putative staphylococcal methyltransferase responsible for this modification (20, 21). These observations suggest that *S. aureus* may employ PCH methylation, which lowers Fe(III)-binding affinity, as a strategy to compete with *P. aeruginosa*. These studies demonstrate that nutrient availability, particularly that of Fe, plays a key role in *P. aeruginosa*/*S. aureus* coculture dynamics.

A study of the metal-sequestering host-defense protein human calprotectin (CP; S100A8/S100A9 heterooligomer, MRP8/MRP14 oligomer) showed that it promotes *S. aureus* survival in coculture with *P. aeruginosa* (22). CP treatment of the coculture caused reduced levels of several anti-staphylococcal factors produced by *P. aeruginosa*, including PQS, the phenazine pyocyanin (PYO), hydrogen cyanide, and HQNO. CP has two transition-metal-binding sites: a His_3_Asp site that sequesters Zn(II) and an unusual hexahistidine (His_6_) metal-binding site that allows it to sequester multiple divalent transition metal ions (23). Based on early studies that reported that CP sequesters Mn(II) and Zn(II) but not other nutrient metal ions (24, 25), enhanced *S. aureus* survival and reduced levels of the anti-staphylococcal metabolites were largely attributed to the ability of CP to sequester Zn(II) and induce Zn-starvation responses in *P. aeruginosa* (22). Nevertheless, it later became evident that the multi-metal sequestration ability of CP extends beyond these two metal ions (26–28). Indeed, just prior to this publication examining the impact of CP on *P. aeruginosa*/*S. aureus* coculture dynamics, CP was first reported to sequester Fe(II) (26), and a period of debate in the community about the biological significance of this finding followed (29–31). Consequently, this initial study on CP and *P. aeruginosa/S. aureus* cocultures did not consider the possibility that Fe(II) sequestration by CP contributed to coculture dynamics and the experimental observations.

Subsequent investigations showed that CP inhibits Fe uptake and elicits Fe-starvation responses in *P. aeruginosa* and *S. aureus* under aerobic conditions (32–34), and that these processes require the His_6_ site. The Fe-starvation responses in *P. aeruginosa* included increased PVD production, upregulation of heme uptake machinery, and induction of the PrrF small regulatory sRNAs that mediate an Fe-sparing response, which reduces dependency on Fe-dependent metabolism (32, 35). Furthermore, as part of a non-canonical Fe-starvation response, CP treatment of *P. aeruginosa* inhibits production of the redox-active phenazines PYO and phenazine-1-carboxylate (PCA) (32), which contribute to many aspects of *P. aeruginosa* pathogenicity including biofilm formation, redox balance, and quorum sensing (36–39). With knowledge that CP lowers Fe availability and induces Fe-starvation responses in *P. aeruginosa*, the observed CP-mediated promotion of *S. aureus* survival in coculture with *P. aeruginosa* was puzzling as the ability of *P. aeruginosa* to kill *S. aureus* was shown to be enhanced under Fe-limited conditions (18, 19).

In this work, we address this apparent dichotomy and examine the consequences of metal deprivation and CP treatment on *P. aeruginosa*/*S. aureus* coculture dynamics. We report that CP elicits robust Fe-starvation responses in both organisms in coculture. Using coculture cell viability assays, we reproduced prior findings and show that while growth of *P. aeruginosa*/*S. aureus* cocultures in Fe-limited medium accelerated *P. aeruginosa* killing of *S. aureus*, CP treatment increased the survival of *S. aureus* grown in coculture with *P. aeruginosa*. Remarkably, by employing CP variants as well as metalated protein in coculture assays, we uncovered that the protective effect of CP on *S. aureus* in coculture is independent of the ability of CP to sequester nutrient metal ions. This advance highlights an underappreciated facet of CP function that is outside of its canonical role in nutritional immunity and underscores the importance of looking beyond its metal-sequestering role in host–pathogen and pathogen–pathogen interactions.

## RESULTS

### Approach for examining the consequences of CP and metal availability on cocultures of *P. aeruginosa* and *S. aureus*

Our experimental design considered prior studies by our laboratory and others that investigated the effects of CP and metal starvation on monocultures and cocultures of *P. aeruginosa* and *S. aureus* (22, 25, 32, 33). For the majority of the coculture studies, we employed *P. aeruginosa* strain UCBPP-PA14 (hereafter PA14) and *S. aureus* strain USA300 JE2 (hereafter JE2). *P. aeruginosa* PA14 is a clinical burn wound isolate commonly employed for studies of *P. aeruginosa* metal homeostasis and virulence (40, 41). It possesses markedly heightened host virulence as compared to PAO1, another commonly employed strain, and its well-studied genome contains virulence genes common to most *P. aeruginosa* strains as well as pathogenicity islands that are not present in PAO1. *S. aureus* JE2 is a stable and prominent strain of community-associated methicillin-resistant *S. aureus* that is commonly used for laboratory studies (42).

We performed the majority of *P. aeruginosa*/*S. aureus* coculture studies in a chemically defined medium (CDM) that was originally described for *S. aureus* growth (43) and that we have employed in prior investigations of both species (32, 33). Metal-replete CDM is prepared from trace metal reagents and select nutrient metals (0.3 µM Mn, 5 µM Fe, 0.1 µM Cu, 0.1 µM Ni, and 6 µM Zn), and includes 1 mM Ca(II) to mimic extracellular Ca(II) levels in the host environment (32). The ability to omit one or more metals from the medium preparation allows studies of *P. aeruginosa* and *S. aureus* growth in controlled metal conditions. In this work, we performed coculture growth studies in Mn-depleted, Fe-depleted, and Zn-depleted CDM as well as metal-depleted CDM (depleted of Mn, Fe, and Zn) (Table S1) to examine the consequences of singlemetal and multi-metal deprivation as compared to CP treatment on coculture growth and *P. aeruginosa* metabolite production. Metal-replete CDM generally served as the untreated control. Excluding one CP titration experiment, all experiments with CP (or variant) were performed using 20 µM protein. This concentration showed an effect in initial coculture growth studies and reflects physiologically relevant CP levels (44, 45). Cocultures were initiated with a 1:2 ratio of *P. aeruginos*a/*S. aureus* colony-forming units (CFUs); the starting inoculum for each species was determined in monocultures by culture turbidity (OD_600_) and CFU counting. Bacterial cultures were grown aerobically at 37°C with shaking at 250 rpm. To monitor the growth of *P. aeruginosa* and *S. aureus* in coculture over time, we determined CFUs at regular intervals by plating aliquots of the coculture on agar selective for either *P. aeruginosa* (*Pseudomonas* isolation agar) or *S. aureus* (Baird-Parker agar).

### CP induces the expression of genes encoding for Fe-starvation responses in both *P. aeruginosa* and *S. aureus* grown in coculture

**Fig 1:**
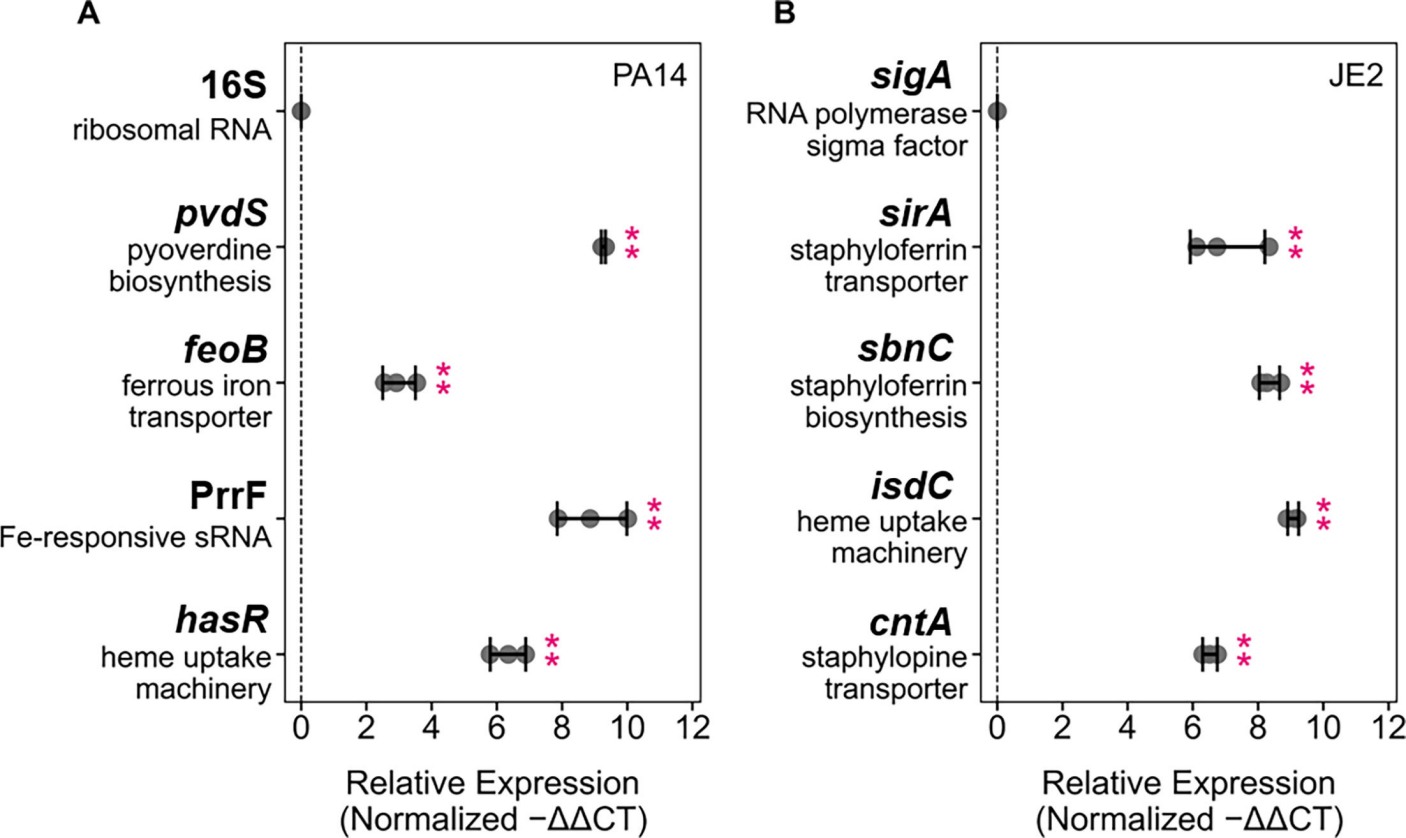
CP induces Fe-starvation responses in both *P. aeruginosa* (**A**) and *S. aureus* (**B**) grown in coculture. Transcript levels were normalized to the respective housekeeping genes (*P. aeruginosa*: 16S; *S. aureus: sigA*), and the fold change after normalization is presented (*n* = 3, **P < 0.01, error bars represent standard deviation [SD]). Cultures were grown in metal-replete CDM ± 20 µM CP and were incubated at 37°C for 6 h.

We first questioned whether CP elicits Fe-starvation responses in *P. aeruginosa* PA14 and in *S. aureus* JE2 grown in coculture. To probe for Fe-starvation responses, we extracted RNA from cocultures of *P. aeruginosa* PA14 and *S. aureus* JE2 grown in the absence and presence of CP. We then employed real-time PCR to quantify expression levels of select genes that participate in hallmark Fe-starvation responses including siderophore biosynthesis and Fe acquisition (Table S2). For *P. aeruginosa*, we examined the expression of *pvdS* (sigma factor that activates the expression of genes for pyoverdine biosynthesis) (46), *feoB* (ferrous Fe transporter) (47), PrrF Fe-responsive sRNAs (35), and *hasR* (heme uptake machinery) (48). For *S. aureus*, we analyzed the expression of *sirA* (staphyloferrin transporter) (49), *sbnC* (staphyloferrin biosynthesis) (50), *isdC* (51) (heme uptake machinery), and *cntA* (staphylopine transporter) (52). In all cases, gene expression was significantly upregulated in *P. aeruginosa*/*S. aureus* cocultures treated with CP as compared to the untreated controls (Fig. 1). These results are consistent with prior studies by our laboratory and others showing that CP induces Fe-starvation responses in monocultures of *P. aeruginosa* and *S. aureus* (32, 33, 53), and demonstrate that CP can induce transcriptional changes consistent with Fe-starvation responses in both *P. aeruginosa* and *S. aureus* grown in coculture.

### CP affects siderophore production by *P. aeruginosa* in coculture with *S. aureus*

To further probe Fe-starvation responses, we examined how CP affects siderophore production by *P. aeruginosa* in monoculture and in coculture with *S. aureus. P. aeruginosa* produces two siderophores, PVD and PCH (54). PVD is thought to be the primary siderophore used by *P. aeruginosa* under conditions of chronic Fe starvation. By contrast, PCH is thought to be representative of an acute or early Fe-starvation response because it is energetically less expensive to produce as compared to PVD (55, 56). We performed time-course studies and quantified levels of PVD and PCH in supernatants obtained from cultures grown in metal-replete CDM with and without CP treatment, as well as Fe-depleted and Zn-depleted CDM. Furthermore, to assess if the metal-binding sites of CP were required for siderophore production by *P. aeruginosa* grown in coculture with *S. aureus*, we examined levels of PVD and PCH at the culture endpoint of cocultures treated with the CP site variants ΔHis_3_Asp, ΔHis_4_, and ΔΔ (32) (Table S3). These variants have multiple amino acid substitutions (His/Asp→Ala) in the transition-metal-binding sites of CP, which make these sites unable to coordinate transition metal ions. The ΔHis_3_Asp variant lacks the Zn(II)-sequestering His_3_Asp site and allows for the multi-metal-sequestering His_6_ site to be probed in isolation, whereas the ΔHis_4_ variant lacks four His of the His_6_ site and enables study of the His_3_Asp site in isolation. The ΔΔ variant lacks both His_3_Asp and His_6_ sites and therefore cannot sequester transition metal ions.

**Fig 2:**
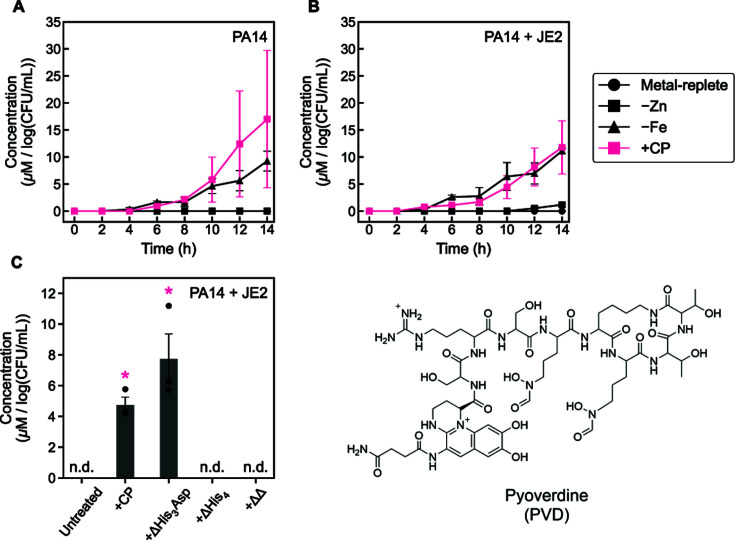
CP treatment increases PVD levels in supernatants from monocultures of *P. aeruginosa* and cocultures of *P. aeruginosa* and *S. aureus*. PVD levels in *P. aeruginosa* monocultures (**A**), in *P. aeruginosa*/*S. aureus* cocultures (**B**), and in *P. aeruginosa*/*S. aureus* cocultures treated with CP site variants (**C**). Cultures were grown in metal-replete CDM ± 20 µM CP (or variant), Zn-depleted CDM, or Fe-depleted CDM, and were incubated at 37°C. Aliquots were collected every 2 h (**A, B**) or at 14 h (**C**) for quantification of metabolites by HPLC. Metabolite levels were normalized to *P. aeruginosa* CFUs (*n* = 3, error bars represent standard error [SE]). See Table S14 for significance testing results for endpoint metabolite levels (**A, B**). For comparison with the untreated culture condition, *P < 0.05 (**C**), n.d. = not detected.

In agreement with prior endpoint studies examining the impact of CP on PVD production (32), negligible PVD was detected in supernatants of *P. aeruginosa* monocultures grown in metal-replete and Zn-depleted CDM, whereas Fe-depleted and CP-treated cultures exhibited a robust increase in PVD levels (Fig. 2A). For the CP-treated and Fe-depleted monocultures, PVD was first detected at 6 h and its levels increased over the remainder of the time-course. PVD levels in *P. aeruginosa/S. aureus* coculture supernatants recapitulated these observations from *P. aeruginosa* monocultures, with negligible PVD detected in the metal-replete and Zn-depleted cultures and PVD first detected at 4 and 6 h for the CP-treated and Fe-depleted cultures, respectively (Fig. 2B). These data show that CP treatment causes PVD production by *P. aeruginosa* in monoculture and coculture with kinetics that are overall comparable to those observed for the cultures grown in Fe-depleted medium. PVD levels in supernatants were also similar for *P. aeruginosa* grown in monoculture or in coculture with *S. aureus*. Evaluation of the CP variants revealed PVD production in *P. aeruginosa*/*S. aureus* cocultures treated with ΔHis_3_Asp but not for cocultures treated with ΔHis_4_ or ΔΔ (Fig. 2C). Taken together, our observations further corroborate that CP induces Fe-starvation responses in *P. aeruginosa* and demonstrate that the presence of a functional His_6_ site in CP is required to induce PVD production in *P. aeruginosa* grown in coculture with *S. aureus*, which is consistent with the ability of this site to sequester Fe(II).

**Fig 3:**
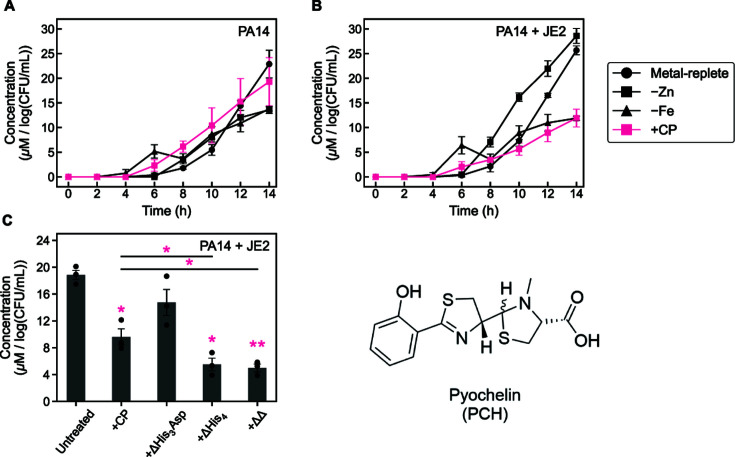
CP treatment impacts PCH levels in supernatants from cocultures of *P. aeruginosa* and *S. aureus*. PCH levels in *P. aeruginosa* monocultures (**A**), *P. aeruginosa/S. aureus* cocultures (**B**), and *P. aeruginosa*/*S. aureus* cocultures treated with CP site variants (**C**). Cultures were grown in metal-replete CDM ± 20 µM CP (or variant), Zn-depleted CDM, or Fe-depleted CDM, and were incubated at 37°C. Aliquots were collected every 2 h (**A, B**) or at 14 h (**C**) for quantification of metabolites by HPLC. Metabolite levels were normalized to *P. aeruginosa* CFUs (*n* = 3, error bars represent SE). See Table S14 for significance testing results for endpoint metabolite levels (**A, B**). For comparison with the untreated culture condition, *P < 0.05, **P < 0.01 (**C**).

By contrast, PCH was detected in the supernatants of *P. aeruginosa* monocultures and cocultures regardless of the medium condition or CP treatment (Fig. 3). For the monocultures, CP treatment and growth in Fe-depleted medium caused earlier initial detection of this metabolite (6 h versus 8 h) (Fig. 3A). The overall rate of increase in PCH levels was slower in both cases relative to production in metal-replete CDM, resulting in slightly lower PCH levels at the culture endpoint for the Fe-depleted and CP-treated cultures. A potential explanation for this observation is that *P. aeruginosa* experiences prolonged Fe starvation when treated with CP or grown in Fe-depleted medium, which causes *P. aeruginosa* to switch from producing PCH to PVD (55, 56). Growth of *P. aeruginosa* monocultures in Zn-depleted medium resulted in a slight decrease in endpoint PCH levels as compared to untreated cultures. The trends in PCH levels for *P. aeruginosa/S. aureus* cocultures were similar to those observed for *P. aeruginosa* monocultures (Fig. 3B): CP treatment or growth in Fe-depleted medium resulted in earlier initial detection of PCH (6 h versus 8 h), an overall slower rate of PCH production, and decreased PCH levels at the culture endpoint as compared to untreated cultures. In contrast to the *P. aeruginosa* monocultures, growth of *P. aeruginosa*/*S. aureus* cocultures in Zn-depleted CDM did not significantly alter endpoint PCH levels. Curiously, the endpoint studies with the CP variants revealed that cocultures treated with ΔΔ or ΔHis_4_ exhibited lower PCH levels than both CP-treated cocultures and the untreated control (Fig. 3C). This surprising finding indicates that further investigation into the structure–function relationship defining how CP modulates PCH production is warranted.

Two recent studies uncovered that PCH can be altered by *S. aureus* via enzymatic methylation of the free carboxylic acid to produce the PCH methyl ester (20, 21). This transformation was also proposed as a mechanism for *S. aureus* competition with *P. aeruginosa* in coculture because methylated PCH exhibits a decreased affinity for Fe(III) as compared to unmodified PCH (20, 21). During our metabolite studies, we detected a minor peak at 21.7 min (Fig. S1) in the high-performance liquid chromatography (HPLC) chromatograms of *P. aeruginosa*/*S. aureus* coculture supernatants (~1% of the total integrated area corresponding to the PCH diastereomer peaks) that eluted shortly after PCH with a *m/z* value of 339.1, which we assigned to be one (or both) of the two diastereomers of methylated PCH (theoretical *m/z* = 339.08, [M+H]^+^ ion). Negligible change in the relative abundance of methylated PCH (as compared to total PCH) was found in the presence of CP or site variants (Fig. S2). Furthermore, the relative abundance of methylated PCH in the presence of ΔΔ resembled that of untreated cultures. Together, these observations show that treatment with CP (or site variants) does not significantly alter the relative abundance of methylated PCH detected in the cocultures. A recent study showed that the *P. aeruginosa*/*S. aureus* ratio in the starting inoculum affected levels of methylated PCH detected in cocultures of *P. aeruginosa* PA14 and *S. aureus* LAC (20). Levels of methylated PCH approximately tripled when the *P. aeruginosa*/*S. aureus* ratio increased from 1:1 to 1:10, and only minor increases in methylated PCH were observed as the proportion of *S. aureus* was increased further. Based on these results, we reason that the relatively low levels of methylated PCH detected in our cocultures are, at least in part, a result of the starting inoculum (1:2 ratio of *P. aeruginosa/S. aureus*).

### CP decreases the production of phenazines by *P. aeruginosa* in coculture with *S. aureus*

**Fig 4:**
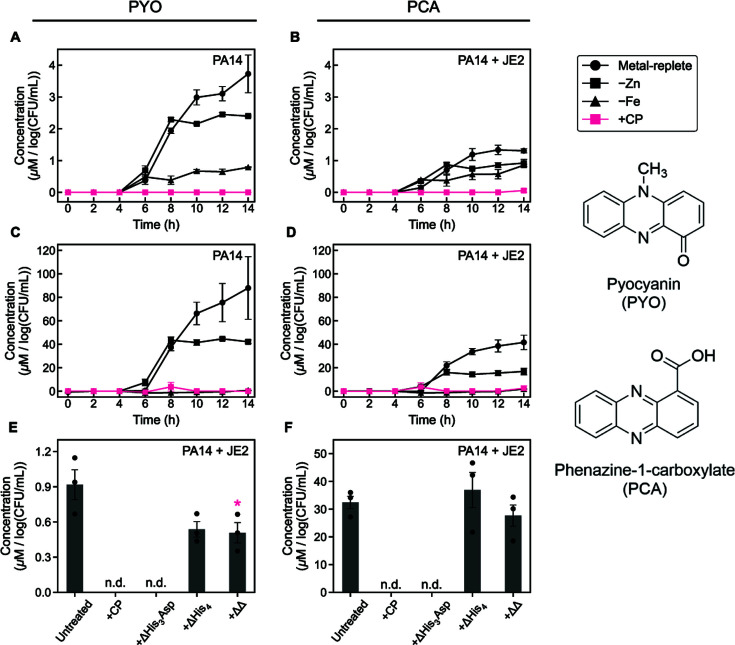
CP treatment suppresses production of the phenazines pyocyanin (PYO) and phenazine-1-carboxylate (PCA). PYO levels in supernatants from *P. aeruginosa* monocultures (**A**) and *P. aeruginosa/S. aureus* cocultures (**B**). PCA levels in supernatants from *P. aeruginosa* monocultures (**C**) and *P. aeruginosa/S. aureus* cocultures (**D**). PYO (**E**) and PCA (**F**) levels in supernatants from *P. aeruginosa*/*S. aureus* cocultures treated with CP site variants. Cultures were grown in metal-replete CDM ± 20 µM CP (or varant), Zn-depleted CDM, or Fe-depleted CDM, and were incubated at 37°C. Aliquots were collected every 2 h (**A–D**) or at 14 h (**E, F**) for quantification of metabolites. Metabolite levels were normalized to *P. aeruginosa* CFUs (*n* = 3, error bars represent SE). See Table S14 for significance testing results for endpoint metabolite levels (**A–D**). For comparison with the untreated culture condition, *P < 0.05 (**E**), n.d. = not detected..

The levels of phenazines in *P. aeruginosa* culture supernatants provide another indicator of Fe starvation. As part of a non-canonical Fe-starvation response, Fe depletion reduces levels of the phenazines PYO and PCA in culture supernatants (32). Thus, we quantified phenazine levels in the same supernatants used for the siderophore analyses. Time-course studies revealed that PYO was first detected at 6 h under all culture conditions that supported its production (Fig. 4A and B). Overall, PYO levels were markedly reduced in *P. aeruginosa*/*S. aureus* coculture supernatants as compared to *P. aeruginosa* monoculture supernatants. Consistent with prior work (22, 32), CP treatment of *P. aeruginosa* monocultures resulted in a near-complete loss of PYO in culture supernatants (Fig. 4A). Furthermore, CP treatment of *P. aeruginosa*/*S. aureus* cocultures resulted in a similar loss of detectable PYO (Fig. 4B). For both monocultures and cocultures, growth in Fe-depleted or Zn-depleted CDM resulted in lower levels of PYO compared to growth in metal-replete CDM, although this effect was lessened in cocultures due to the overall decrease in PYO levels. Overall trends in PCA levels (Fig. 4C and D) closely mirrored those of PYO, with considerably reduced levels of PCA detected in coculture supernatants as compared to those from monocultures. In agreement with prior studies (32), CP treatment of *P. aeruginosa* monocultures resulted in negligible PCA detected (Fig. 4C), an observation that extended to cocultures (Fig. 4D). Unlike PYO, growth in Fe-depleted CDM resulted in minimal PCA production in both monocultures and cocultures. Thus, CP treatment reduces phenazine production in both monocultures and cocultures, and this effect appears to be mediated by metal sequestration.

Because coculture growth in Fe- and Zn-depleted media resulted in attenuated phenazine levels, we next investigated how the metal-binding sites of CP contribute to this effect. Analysis of endpoint supernatants obtained from cocultures treated with the three CP site variants revealed that ΔHis_3_Asp treatment completely abrogated PYO and PCA production by *P. aeruginosa* in coculture (Fig. 4E and F), indicating that the His_3_Asp site is not required for the effect of CP on phenazine levels. By contrast, treatment with ΔHis_4_ or ΔΔ resulted in modest decreases in PYO levels and negligible change in PCA levels as compared to the untreated control (Fig. 4E and F). These data indicate that the His_6_ site of CP is required to fully reduce PYO and PCA levels in these cocultures. Taken together, our results demonstrate that CP effectively suppresses phenazine production by *P. aeruginosa* in coculture with *S. aureus*. In addition, our findings agree with prior studies of *P. aeruginosa* highlighting the importance of Fe availability for the production of PYO and PCA by *P. aeruginosa* (32), and extend these observations to *P. aeruginosa* grown in coculture with *S. aureus*. The modest decrease in endpoint PYO production resulting from treatment of the cocultures with either ΔΔ or ΔHis_4_ points to the ability of CP to decrease PYO production in the absence of the metal-binding sites. This observation warrants further investigation.

### CP-mediated survival of *S. aureus* in *P. aeruginosa/S. aureus* cocultures cannot be attributed to metal depletion

**Fig 5:**
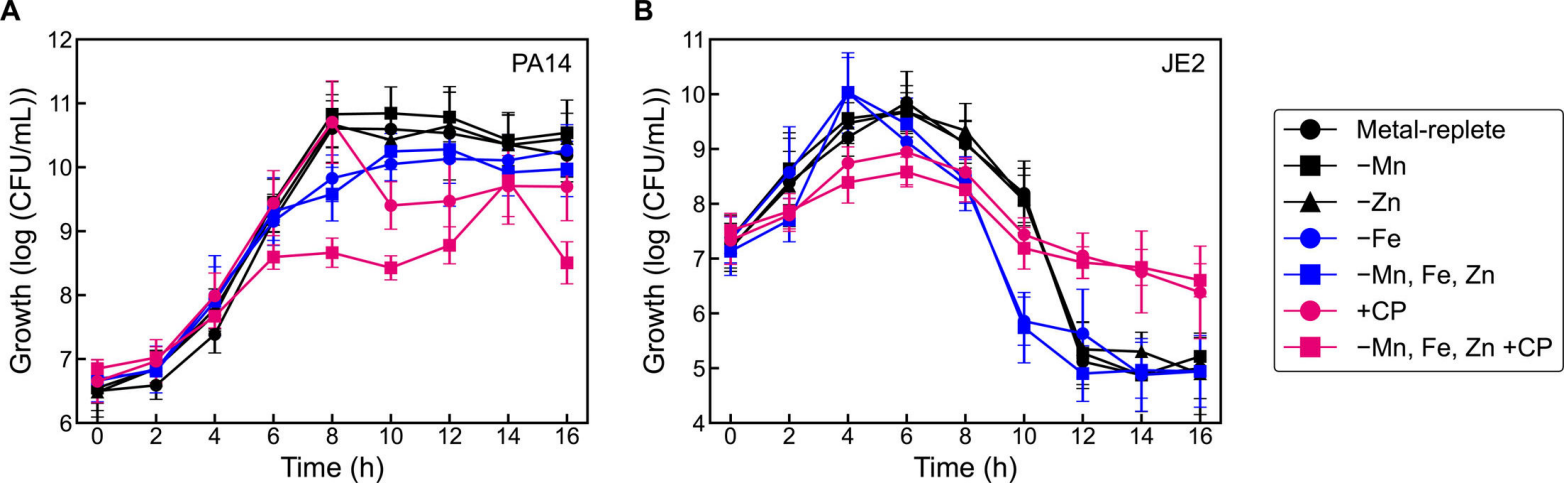
Effect of CP and metal depletion on *P. aeruginosa* (**A**) and *S. aureus* (**B**) growth in coculture. Cocultures were grown in metal-replete CDM, single metal-depleted CDM, or metal-depleted CDM ± 20 µM CP, and were incubated at 37°C (*n* ≥ 3; error bars represent SE). See Table S15 for significance testing results for growth at the 10–16 h timepoints.

Our initial growth studies in metal-replete CDM revealed that CP promoted the survival of *S. aureus* JE2 in coculture with *P. aeruginosa* PA14 (Fig. 5), an observation that was consistent with a previous report (22). However, this result was seemingly inconsistent with other prior studies showing that Fe deprivation enhances *P. aeruginosa* killing of *S. aureus* (18, 19). To ascertain whether this consequence of CP treatment was strain-dependent, we performed coculture growth studies using *P. aeruginosa/S. aureus* strain combinations including PAO1 with JE2, the CF clinical isolate *P. aeruginosa* JSRI-1 with JE2, PA14 with *S. aureus* Newman, and PA14 with *S. aureus* COL. In all cases, CP treatment promoted *S. aureus* survival during coculture with *P. aeruginosa*, with some attenuation of the survival effect seen for the JSRI-1/JE2 combination (Fig. S3 to S6). To probe the effect of different medium conditions, we repeated the PA14/JE2 growth assay in Tris:tryptic soy broth (TSB) supplemented with Ca(II), a medium that has been commonly used in studies examining how metal sequestration by CP impacts microbial pathogens (22, 32, 57, 58), and observed that CP treatment enhanced *S. aureus* survival in this medium (Fig. S7). We also varied the CP concentration (0–40 μM) and found that *S. aureus* survival correlated with CP concentration; higher CP concentrations enhanced *S. aureus* survival relative to lower CP concentrations (Fig. S8). Collectively, these results agree with a prior report showing that CP promotes *S. aureus* survival in coculture with *P. aeruginosa* (22), and demonstrate this observation holds for multiple strains of each species and different media preparations.

Next, we examined the consequences of CP treatment in comparison to nutrient metal depletion for *P. aeruginosa* and *S. aureus* in coculture by monitoring the viability of each species when grown in metal-replete (±CP), Mn-depleted, Fe-depleted, Zn-depleted, and metal-depleted (±CP) CDM (Fig. 5). Strikingly, the growth kinetics data for *S. aureus* in these cocultures clustered into three distinct groups (Fig. 5B), while no significant difference in the viability of *S. aureus* monocultures was observed across the conditions tested (Fig. S9). The first group included cocultures grown in metal-replete, Mn-depleted, and Zn-depleted CDM (Fig. 5B, black lines). *S. aureus* growth in Mn-depleted and Zn-depleted CDM resembled that in metal-replete CDM, with a majority of *S. aureus* killing by *P. aeruginosa* occurring between 10 and 12 h. The second group was defined by cultures grown in Fe-depleted and metal-depleted CDM (Fig. 5B, blue lines). In agreement with prior reports (18, 19), *S. aureus* killing by *P. aeruginosa* was accelerated in Fe-depleted CDM and metal-depleted CDM (blue lines), occurring mostly between 8 and 10 h. The CP-treated cultures constituted the third group and showed a markedly attenuated decline in *S. aureus* killing (Fig. 5B, pink lines). At the 12 h timepoint, *S. aureus* cell viability was 10^2^–10^3^ higher for cocultures that had been treated with CP regardless of the metal content of the medium.

**Fig 6:**
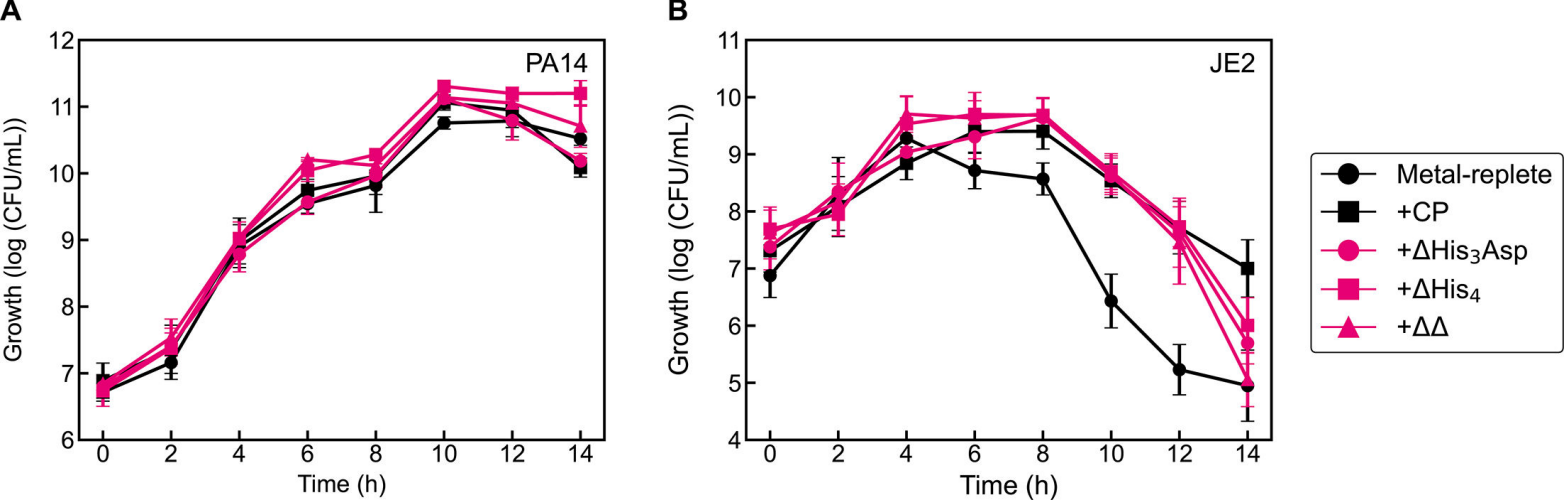
Effect of CP site variants on *P. aeruginosa* (**A**) and *S. aureus* (**B**) growth in coculture. Cultures were grown in metal-replete CDM ± 20 µM CP (or variant) and were incubated at 37°C (*n* ≥ 3; error bars represent SE). See Table S16 for significance testing results for growth at the 10–14 h timepoints.

Although *P. aeruginosa* growth in the coculture was overall similar regardless of condition, we noted the growth curves could be clustered into the same three groups defined by *S. aureus* (Fig. 5A). Growth in Mn-depleted or Zn-depleted CDM had negligible effect on *P. aeruginosa* viability as compared to the metal-replete control (Fig. 5A, black lines), whereas cultures grown in Fe-depleted and metal-depleted CDM (Fig. 5A, blue lines) showed that overall *P. aeruginosa* viability entering stationary phase (8–10 h) was decreased by about a factor of 10 (1 log [CFU/mL]) compared to the metal-replete condition. Again, CP-treated cultures constituted the third group (Fig. 5A, pink lines) and exhibited the lowest overall viability of *P. aeruginosa* by the culture endpoint (16 h). Similar trends in growth were observed for *P. aeruginosa* monocultures (Fig. S9).

**Fig 7:**
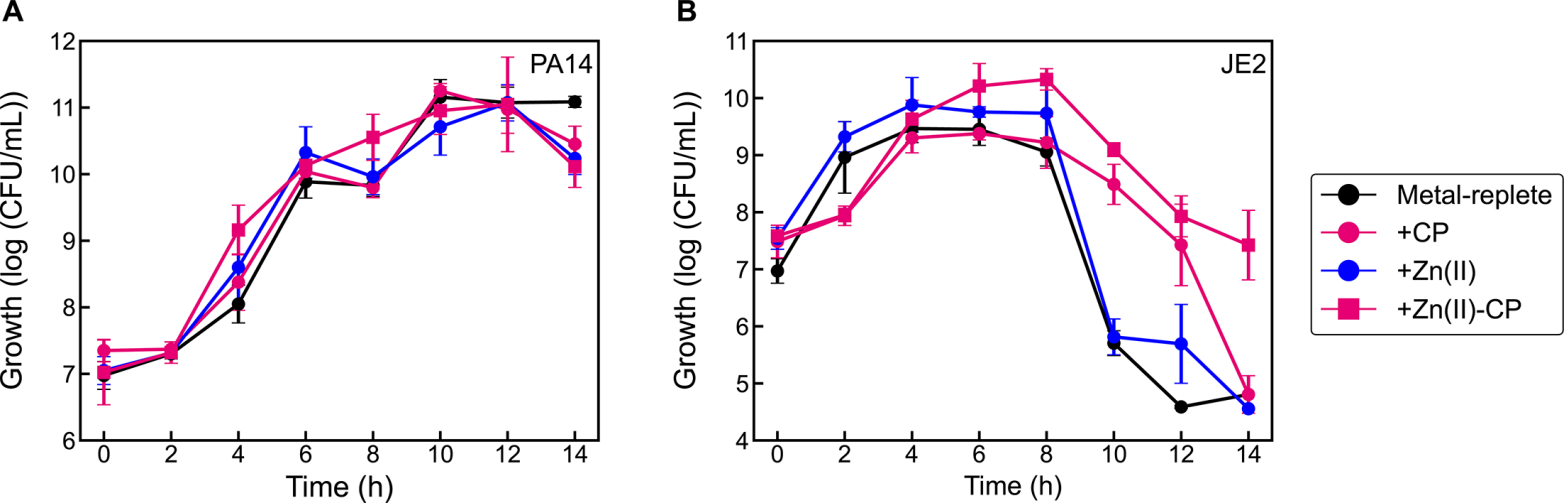
Effect of Zn(II)-bound CP on *P. aeruginosa* (**A**) and *S. aureus* (**B**) growth in coculture. Cultures were grown in metal-replete CDM treated with 20 µM CP, 40 µM Zn(II), or 20 µM Zn(II)-bound CP, and were incubated at 37°C (*n* ≥ 3; error bars represent SE). See Table S16 for significance testing results for growth at the 10–14 h timepoints.

We considered the possibility that CP treatment might act by decreasing *P. aeruginosa* viability during stationary phase and thus contribute to increased *S. aureus* survival. However, a comparison of *P. aeruginosa* and *S. aureus* growth during coculture revealed that reduced *S. aureus* CFUs do not correlate with increased *P. aeruginosa* CFUs. Moreover, subsequent coculture experiments demonstrated that there is some degree of variability in *P. aeruginosa* growth between biological replicates and experiments (e.g., Fig. 6, 7 and 9), which may arise from the inherent variability associated with studying competitive bacterial cocultures. We note that the effect of CP promoting *S. aureus* survival in coculture with *P. aeruginosa* occurs consistently even when *P. aeruginosa* viability in CP-treated cultures resembles untreated cultures, indicating that the effect of CP is not driven by changes in *P. aeruginosa* viability. Collectively, these results illuminate the remarkable and opposing effects of Fe starvation and CP treatment on *S. aureus* growth in coculture with *P. aeruginosa*. Despite CP being a Fe(II)-sequestering protein that has the ability to induce Fe-starvation responses in *P. aeruginosa* in both monoculture and coculture, it promotes *S. aureus* survival in cocultures of *P. aeruginosa* and *S. aureus*.

### CP-mediated survival of *S. aureus* in coculture does not require the metal-binding sites of CP

To further probe whether metal sequestration by CP contributed to its ability to enhance *S. aureus* survival in coculture with *P. aeruginosa*, we expanded the coculture growth studies to the three CP variants ΔHis_3_Asp, ΔHis_4_, and ΔΔ. Comparable *S. aureus* survival was observed following treatment of the cocultures with CP and each variant (Fig. 6), although we observed variability in *S. aureus* survival at the culture endpoint. Thus, the His_3_Asp and His_6_ metal-binding sites of CP are not essential for the protein to promote *S. aureus* survival in coculture with *P. aeruginosa*.

Because two prior studies proposed that Zn(II) sequestration by CP results in decreased killing of *S. aureus* by *P. aeruginosa* in coculture (22, 59), and one investigation reported that Zn(II) supplementation attenuated the effect of CP treatment on the coculture (22), we also evaluated the consequences of treating *P. aeruginosa/S. aureus* cocultures with Zn(II)-bound CP. We prepared Zn(II)-bound CP by pre-incubating CP with two equivalents of Zn(II) and treated the coculture with the resulting complex. In parallel, we also supplemented cocultures with the equivalent amount of Zn(II). Treatment of the coculture with Zn(II)-bound CP had a negligible effect on *P. aeruginosa* viability yet promoted *S. aureus* survival compared to the untreated control (Fig. 7). Moreover, Zn(II) supplementation had negligible effects on *P. aeruginosa* or *S. aureus* growth in coculture as compared to the untreated control (Fig. 7). Our result with Zn(II)-bound CP is in disagreement with a prior study reporting that addition of two equivalents of Zn(II) to cocultures treated with CP fully restored the ability of *P. aeruginosa* to kill *S. aureus* (22). Although the origin of this discrepancy is unclear, Zn(II) addition to solutions of CP can cause the protein to precipitate. One potential explanation for the different results is that Zn(II)-induced precipitation of the protein occurred under the conditions used in the prior work. Together, our data uncover that the ability of CP to enhance *S. aureus* survival in coculture with *P. aeruginosa* is independent of its metal-sequestering function. This effect requires neither the His_3_Asp nor the His_6_ site and cannot be attributed to sequestration of one or all of the nutrient metals Mn(II), Fe(II), and Zn(II).

**Fig 8:**
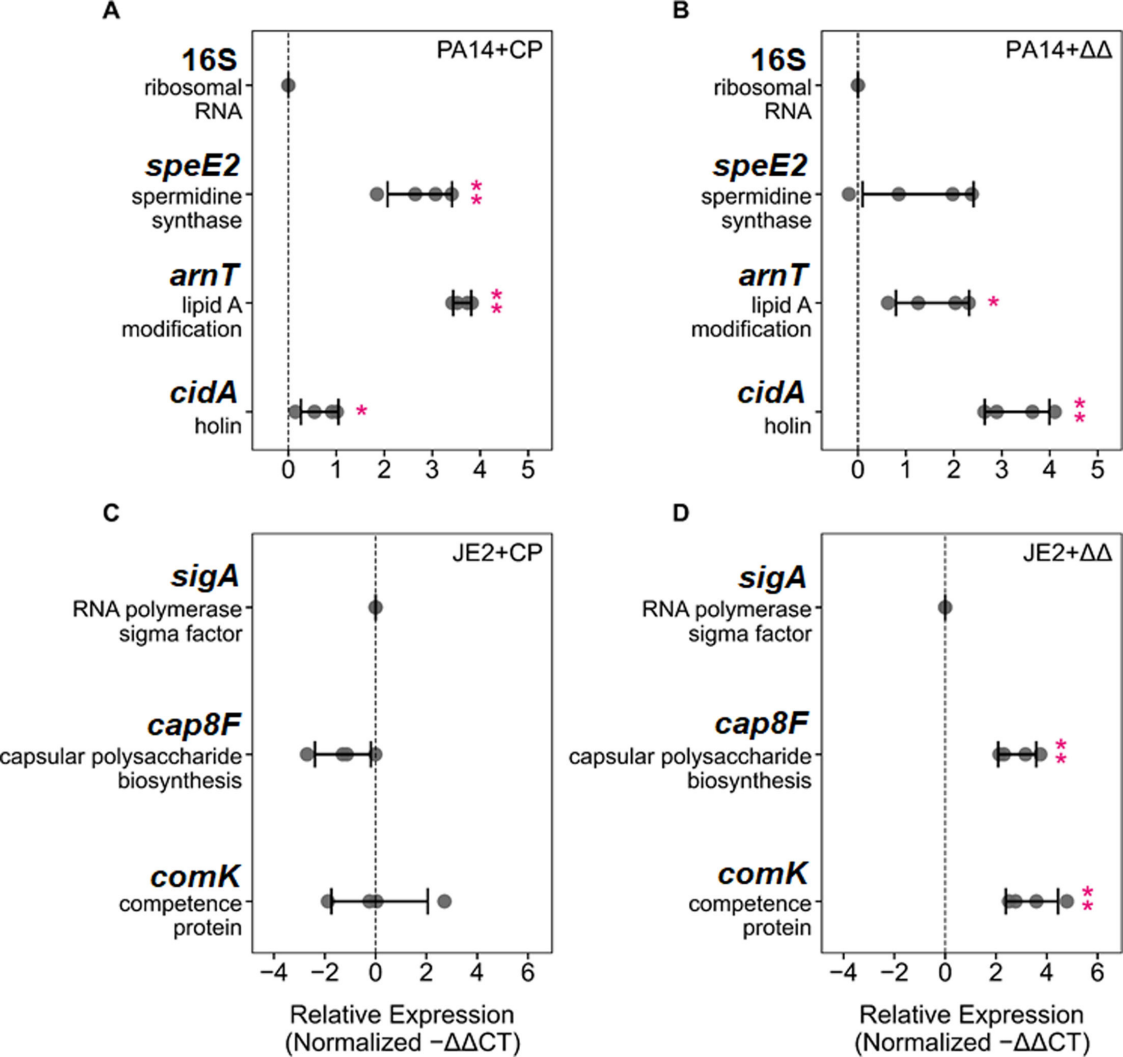
The CP protein scaffold perturbs the expression of genes that affect the cell envelopes of *P. aeruginosa* (A,B) and *S. aureus* (C,D) in coculture. Treatment with CP (**A**) and ΔΔ (**B**) induced membrane modifications for *P. aeruginosa* in coculture. While CP (**C**) did not significantly impact the expression of the genes associated with membrane modifications for *S. aureus* in coculture, treatment with ΔΔ (**D**) resulted in robust upregulation. Transcript levels were normalized to the respective housekeeping genes (*P. aeruginosa*: 16S; *S. aureus: sigA*), and the fold change after normalization is presented (*n* = 4, **P < 0.01, *P < 0.05, error bars represent SD). Cultures were grown in metal-replete CDM ± 20 µM CP and were incubated at 37°C for 6 h.

This metal-independent effect of CP reminded us of our earlier proteomics study of *P. aeruginosa* in which CP treatment upregulated proteins associated with membrane remodeling and tolerance to cationic antimicrobial peptides (60). These upregulated proteins included several proteins encoded by the *arn* operon (lipid A modification) (60, 61) and the spermidine synthase SpeE2 (60, 62). These results suggested that CP treatment impacts the integrity of the *P. aeruginosa* cell envelope. These expression changes were not attributable to the depletion of Fe, Mn, Zn, or all three metals from the culture medium (60). We therefore questioned whether CP perturbs the cell envelope of *P. aeruginosa* in coculture. We found that CP treatment upregulated the expression of *arnT* and *speE2* by *P. aeruginosa* in coculture with *S. aureus* (Fig. 8A). Moreover, ΔΔ treatment increased the expression of *arnT* by *P. aeruginosa* in coculture (Fig. 8B), indicating that the CP protein scaffold induces membrane remodeling. In addition, intrigued by prior findings that CP increased extracellular DNA (eDNA) levels in *P. aeruginosa* monocultures (63), we reasoned that CP or ΔΔ might also affect the release of eDNA by *P. aeruginosa* in coculture. *P. aeruginosa* possesses multiple holins, including CidA (PA3432), which contribute to the release of extracellular DNA via endolysin-mediated cell lysis (64–68). CidA has been shown to be required for the sustained release of eDNA (65), but the mechanisms through which CidA mediates endolysin transport and release in *P. aeruginosa* are not well understood. We observed that both CP and ΔΔ treatment increased the expression of *cidA* (Fig. 8A and B), indicating that the CP protein scaffold induces genes encoding for proteins that mediate the release of eDNA by *P. aeruginosa* in coculture. While the effects of CP on these select genes were largely recapitulated in *P. aeruginosa* monocultures (Fig. S10A), the effects of ΔΔ were seen only for *P. aeruginosa* in coculture (Fig. 8B; Fig. S10B).

CP was also reported to perturb the cell membrane of *S. aureus*, with RNA-seq experiments revealing altered capsule production and membrane competence caused by CP treatment (53, 69, 70). These observations motivated us to question whether the cell envelope of *S. aureus* is also perturbed by CP treatment in coculture. We examined the effects of CP and ΔΔ treatment on the expression of *cap8F* (capsular polysaccharide biosynthesis) (71, 72) and *comK* (competence) (73) in *S. aureus* (Fig. 8C and D). We observed that ΔΔ treatment resulted in marked upregulation of *cap8F* and *comK* (Fig. 8D) by *S. aureus* in coculture with *P. aeruginosa*. By contrast, *S. aureus* in CP-treated cocultures (Fig. 8C) did not exhibit significant upregulation of these target genes. In monocultures, treatment with ΔΔ resulted in no significant upregulation of these genes (Fig. S10D), and CP treatment decreased the expression of *cap8F* (although not statistically significant) and *comK*. Collectively, our findings highlight the capacity of the CP protein scaffold to perturb the cell envelopes of both *P. aeruginosa* and *S. aureus* in coculture, independent of the metal-sequestering ability of the protein.

### Increased survival of *S. aureus* in *P. aeruginosa/S. aureus* cocultures does not require the S100A9 C-terminal tail

**Fig 9:**
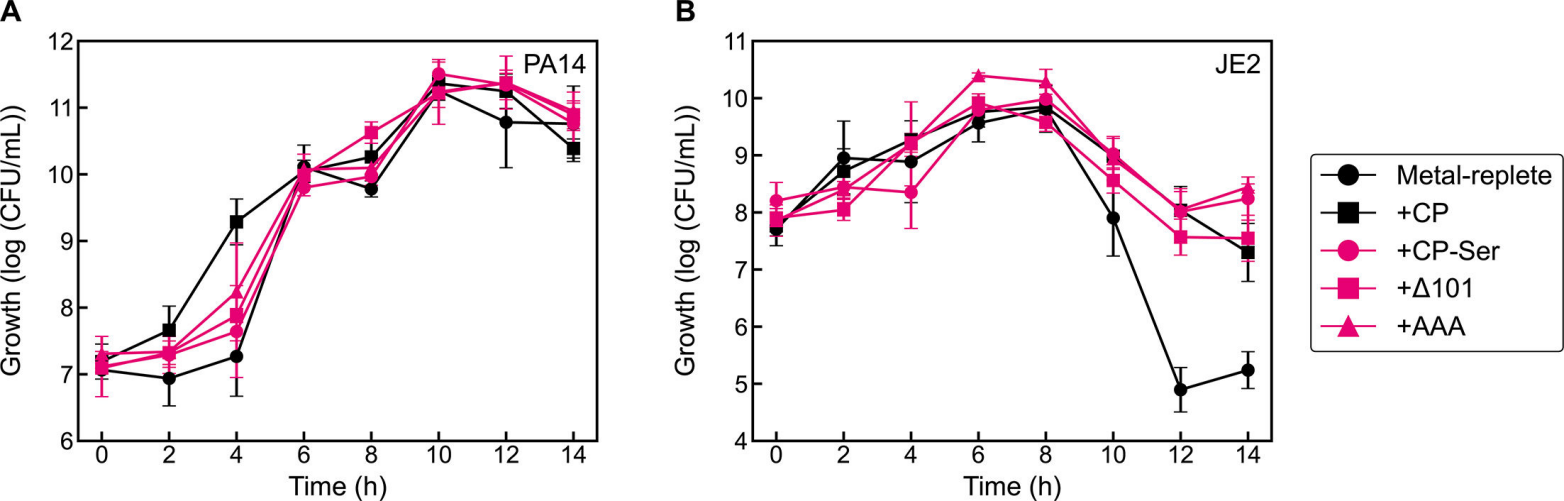
Effect of CP-Ser and S100A9 C-terminal tail variants Δ101 and AAA on *P. aeruginosa* (**A**) and *S. aureus* (**B**) growth in coculture. Cultures were grown in metal-replete CDM ± 20 µM CP, (or variant) and were incubated at 37°C (*n* = 3; error bars represent SE). See Table S16 for significance testing results for growth at the 10–14 h timepoints.

We next questioned if the S100A9 C-terminal tail contributes to the ability of CP to mediate *S. aureus* survival. This region, defined as residues 96-114, is dynamic in the absence of a metal ion bound to the His_6_ site. It plays a critical role in metal sequestration because it houses two His residues of the His_6_ site (H103 and H105) and encapsulates the bound metal ion (25, 74, 75). Beyond metal sequestration, several studies have shown that the S100A9 C-terminal tail binds fatty acids including arachidonic acid and oleic acid (76–79). Thus, we wondered whether the flexible S100A9 tail might provide a binding pocket for capturing antimicrobials, anti-staphylococcal factors, or other small molecules (such as quorum sensing molecules) that could influence coculture dynamics. We examined two available tail variants, AAA and Δ101 (Table S3) (74). The AAA variant has H103-H104-H105 substituted by a tri-Ala sequence and was originally prepared to interrogate the contribution of these residues to metal binding at the His_6_ site. The Δ101 variant is a truncation lacking residues 102-114 of S100A9. Because the available AAA and Δ101 variants were derived from CP-Ser, a CP variant that has its only two Cys residues substituted by Ser residues [S100A8(C42S)/S100A9(C3S) variant], we also evaluated CP-Ser in this assay. Similar to CP, we observed that the CP-Ser and the tail variants had negligible effect on *P. aeruginosa* viability, and the three proteins conferred CP-like protection to *S. aureus* in coculture with *P. aeruginosa* (Fig. 9). These results demonstrate that the C-terminal tail of S100A9 is not essential for the protective effect of CP on *S. aureus* in coculture with *P. aeruginosa*. In addition, these assays show that the two Cys residues in CP are not essential for the activity of CP in *P. aeruginosa*/*S. aureus* cocultures.

### CP heterooligomer is more effective at mediating *S. aureus* survival than S100 homodimers

**Fig 10:**
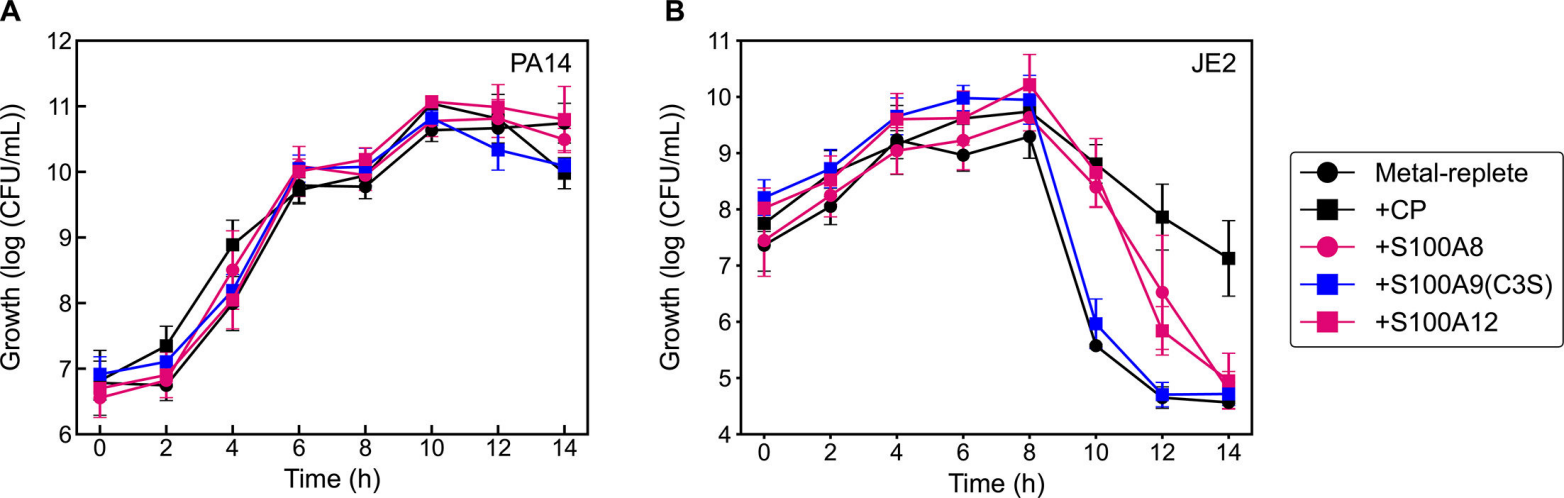
Effect of S100 proteins on *P. aeruginosa* (**A**) and *S. aureus* (**B**) growth in coculture. Cultures were grown in metal-replete CDM ± 20 µM protein and were incubated at 37°C (*n* = 3; error bars represent SE). See Table S16 for significance testing results for growth at the 10–14 h timepoints.

To gain insight into whether the protective effect of CP could be attributed to the heterooligomer or to one of its constituent subunits, we tested the ability of the human S100A8 and human S100A9 homodimers to confer protection of *S. aureus* in coculture with *P. aeruginosa*. We isolated the S100A8 homodimer from a CP reconstitution and purification conducted with an excess of the S100A8 subunit (Supplemental Information). We used the S100A9(C3S) variant, which we previously reported, to investigate the S100A9 homodimer (74). *S. aureus* viability in cocultures treated with S100A9(C3S) resembled that of the untreated cultures (Fig. 10). By contrast, the S100A8 homodimer conferred a protective effect to *S. aureus*, albeit attenuated compared to that conferred by CP (Fig. 10). The cocultures treated with S100A8 exhibited more rapid *S. aureus* killing at *t* > 10 h compared to those treated with CP. We also evaluated human S100A12, a homodimer that sequesters Zn(II) (80, 81), in parallel and found it exerted similar effects on *S. aureus* growth as S100A8. These three proteins had negligible effect on *P. aeruginosa* growth in coculture with *S. aureus* (Fig. 10). Our observations suggest that the S100A8 subunit of CP plays a role in the activity of CP in *P. aeruginosa*/*S. aureus* cocultures yet highlight that the heterooligomeric structure of CP is important for the protective effect toward *S. aureus*.

**Fig 11:**
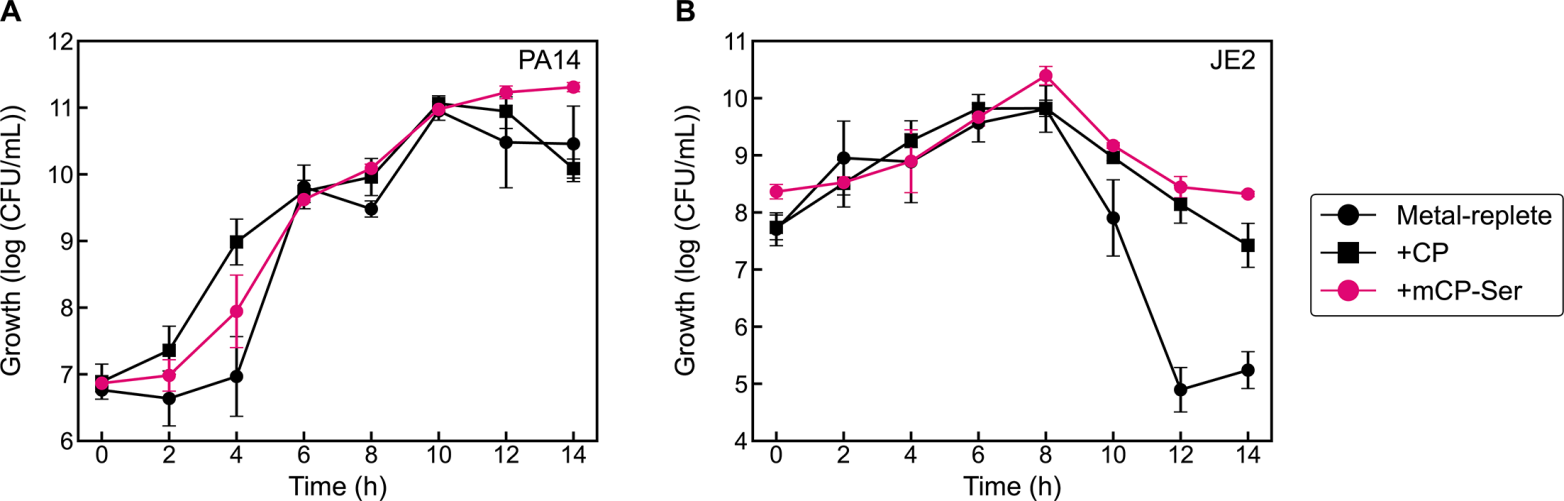
Effect of murine CP-Ser on *P. aeruginosa* (**A**) and *S. aureus* (**B**) growth in coculture. Cultures were grown in metal-replete CDM ± 20 µM CP or mCP-Ser and were incubated at 37°C (*n* = 3; error bars represent SE). See Table S16 for significance testing results for growth at the 10–14 h timepoints.

Lastly, we questioned whether the ability of CP to mediate *S. aureus* survival was specific to the human protein and thus considered the consequence of treating *P. aeruginosa/S. aureus* cocultures with murine CP (mCP), which is a heterooligomer of murine S100A8 and murine S100A9. CP and murine CP-Ser (the Cys-null variant of murine CP) (82) displayed comparable impact on *P. aeruginosa* and *S. aureus* growth in coculture, revealing that the ability of CP to promote *S. aureus* survival is shared by the human and murine proteins (Fig. 11). This observation is intriguing given that human and murine CP share only ~56% amino acid identity (82).

## DISCUSSION

In this work, we explored the apparent dichotomy between the effects of Fe starvation and CP treatment on *S. aureus* growth in *P. aeruginosa*/*S. aureus* cocultures. We leveraged prior studies on *P. aeruginosa* and *S. aureus* monocultures and experimental tools that included defined media conditions with controlled metal levels and CP site variants (32, 33). Our results demonstrate that CP elicits Fe-starvation responses in both *P. aeruginosa* and *S. aureus* grown in coculture. In agreement with prior coculture studies, we found that Fe deprivation enhances *S*. a*ureus* killing by *P. aeruginosa* and that CP promotes *S. aureus* survival in coculture with *P. aeruginosa*. Thus, in the presence of CP, it appears that *S. aureus* survival outcomes in coculture with *P. aeruginosa* are not dictated by Fe availability or Fe starvation but are instead governed by additional activities of CP that require elucidation. Moreover, our data demonstrate that this protective effect of CP toward *S. aureus* does not require its metal-sequestering activity. This metal-independent effect of CP on *P. aeruginosa*/*S. aureus* cocultures falls outside of the canonical role of the protein in nutritional immunity.

Over the past two decades, most studies investigating the effects of CP on microbial physiology have focused on the consequences of its metal-sequestering function. There are, however, limited examples indicating metal-independent activities of CP relevant to the host–pathogen interaction (30, 60, 83–86). A subset of these studies, performed using monocultures, revealed impacts of CP on bacterial membranes (60, 83). For instance, CP was reported to bind to the membrane of the Lyme disease pathogen *Borrelia burgdorferi* and sensitize this membrane to osmotic stress, an effect that was found to be independent of its metal-withholding ability (83). Our prior proteomics study on *P. aeruginosa* revealed that CP treatment increased levels of membraneremodeling proteins (60). Changes in the levels of these proteins were not attributable to metal depletion (Mn, Fe, Zn) (60), suggesting that CP affects the *P. aeruginosa* cell envelope in a way that does not involve metal sequestration. Indeed, our current studies demonstrate that ΔΔ increases the expression of genes involved in membrane remodeling and the release and uptake of eDNA for both *P. aeruginosa* and *S. aureus* in coculture, further indicating that CP perturbs the cell envelopes of both organisms in coculture in a metal-independent manner. The results also show that ΔΔ had a stronger effect than CP on the *S. aureus* genes tested. This observation requires further investigation, but one plausible explanation is that the metal-sequestering activity of CP masks metal-independent effects of the protein scaffold on *S. aureus*. This important finding demonstrates the need to consider how metal availability, as well as the metal-dependent and metal-independent activities of CP, modulate the transcriptional responses of each organism to one another.

Additional investigations that point to CP treatment impacting bacterial membranes include an RNA-seq study of *S. aureus*, which showed that CP treatment upregulated the expression of genes involved in the modification of cell wall teichoic acids (53), as well as a report demonstrating that CP treatment induces lipid A modifications in *Helicobacter pylori* (87). Collectively, these observations and our results indicate that CP treatment has consequences on the chemical composition and stability of membranes from both gram-negative and gram-positive bacterial pathogens. While some studies indicate these effects are independent of metal sequestration (60, 83), others indicate that metals play a role (53, 87). It will be important to distinguish between effects of CP on the cell envelope involving metal sequestration or the metal-binding sites of CP, versus effects that do not involve metal sequestration by using media conditions with controlled metal concentrations and CP site variants that do not bind transition metal ions. These future studies are particularly important given what we have shown here: that the effects of CP treatment of *P. aeruginosa/S. aureus* cocultures differ significantly from that of metal depletion.

CP was also reported to increase the expression of the *P. aeruginosa* two-component system PmrAB (88) in a manner independent of metal depletion (60). PmrAB is known to be upregulated in response to cationic antimicrobial peptides (89, 90) as well as changes in surface charge due to increased levels of extracellular DNA (eDNA), an important component of the *P. aeruginosa* extracellular polymeric substance (EPS) (91). Here, we further show that ΔΔ increases the expression of a *S. aureus* competence gene that mediates eDNA uptake (*comK*). Combined, these findings suggest that both pathogens modulate levels of genes related to eDNA uptake (*cidA* for *P. aeruginosa*, *comK* for *S. aureus*) and sensing (*pmrAB* for *P. aeruginosa*) in response to the CP protein scaffold. It is currently unclear which structural features of the CP protein scaffold are responsible for this activity, and the implications of these findings for interactions between *P. aeruginosa* and *S. aureus* require elucidation. One recent study of *P. aeruginosa*/*S. aureus* cocultures showed that treatment of the coculture with CP resulted in increased carbohydrate content within the EPS of the *P. aeruginosa*/*S. aureus* biofilm matrix (63). The same study also revealed a previously unappreciated effect of CP: CP treatment of *P. aeruginosa*/*S. aureus* cocultures led to the formation of fibrillar, mesh-like structures, which encased *P. aeruginosa* and *S. aureus* cells (63). Formation of the mesh-like structure is reminiscent of human α-defensin 6, a small peptide that forms “nanonets” that entrap bacterial pathogens (92, 93). Indeed, it is possible that this mesh-like structure, changes in EPS content, and/or perturbations to cell envelope surface-associated charge promote *S. aureus* survival. It will be informative to decipher whether metal ions contribute to these phenomena.

The ΔΔ variant is one tool for studying metal-independent effects of CP. Prior microbiological studies with the ΔΔ variant that extend beyond growth assays are relatively limited but have hinted at potential metal-independent effects of CP (30, 84, 85). One study seeking to model expression profiles of *P. aeruginosa* PAO1 within a bacterial infection model identified 10 unique genes describing the response of PAO1 to ΔΔ in a synthetic CF medium (86). Our observation that ΔΔ treatment decreased levels of PYO and PCH in *P. aeruginosa*/*S. aureus* cocultures indicates that the ΔΔ variant—and hence the CP protein scaffold alone—affects bacterial metabolism. Understanding how CP affects pathogens in a metal-independent manner is a ripe subject of future investigative efforts.

In closing, this contribution provides a foundation for future exploration on several fronts. First, how CP impacts interspecies interactions between *P. aeruginosa* and *S. aureus* requires further evaluation. Whether the CP-mediated protective effect on *S. aureus* results from decreased virulence of *P. aeruginosa*, increased fitness of *S. aureus*, or some other phenomena requires elucidation. Second, the vast majority of investigations of CP reported to date were performed using monocultures, and further investigation of CP in other more complex systems including cocultures and polymicrobial communities is likely to further reveal novel mechanisms by which CP contributes to innate immunity and impacts interspecies interactions. Third, this work focuses attention to the fact that CP has metal-independent roles in the host–pathogen and pathogen–pathogen interactions. The current working model for how CP contributes to immunity and the host–pathogen interaction focuses on nutritional immunity and its versatile metal-sequestering ability. We look forward to further deciphering its metal-independent impact on *P. aeruginosa/S. aureus* cocultures, identifying other underappreciated metal-independent effects of CP, and integrating these findings into our current understanding of this remarkable protein.

## MATERIALS AND METHODS

For complete materials and methods, please refer to the accompanying Supplemental Information.

### Solutions, buffers, and metal stocks

All chemicals and reagents were purchased from commercial vendors and were used as received. All solutions were made using Milli-Q water (18.2 MΩ·cm, Milli-Q Academic system). All buffers and metal stocks were filtered (0.2 µm) before use. All solutions and buffers used for microbiology were prepared using metal-free or trace metals basis reagents. Stock solutions of metal salts were prepared by dissolving high-purity metal salts (Sigma) in Milli-Q water using acid-washed volumetric glassware. The stock solutions (Ca, Mg, Mn, Ni, Cu, Zn) were then stored in polypropylene tubes. The following metal salts were used: CaCl_2_·2H_2_O (99.0% BioReagent), MgSO_4_ (99.99%), MnCl_2_·4H_2_O (99.99%), NiSO_4_·6H_2_O (99.99%), CuSO_4_·5H_2_O (99.999%), and anhydrous ZnCl_2_ (99.999%). Iron stock solutions were prepared immediately before use by dissolving (NH_4_)_2_Fe(SO_4_)_2_·6H_2_O (99.997%) in Milli-Q water under ambient conditions.

### Bacterial strains and commercial growth media

The full list of bacterial strains utilized in this study is shown in Table S8. All growth media with the exception of CDM were purchased from commercial vendors. Luria-Bertani (LB, Miller) medium (BD Difco), tryptic soy broth (BD Difco), *Pseudomonas* Isolation Agar (Sigma, BD Difco), and Baird-Parker medium (Sigma, BD Difco) were dissolved in Milli-Q water, sterilized, and prepared according to the manufacturer’s recommendations. Sterilized Baird-Parker medium was completed by the addition of egg yolk tellurite emulsion (Sigma, BD Difco). Tris:TSB medium was prepared by mixing sterilized antimicrobial activity (AMA) buffer (20 mM Tris, 100 mM NaCl, pH 7.5) and TSB in a 62:38 ratio and supplemented with 2 mM of Ca(II) immediately before use.

### Protein overexpression, purification, and handling

CP (S100A8/S100A9 heterodimer), CP variants, S100A9(C3S), S100A12, and mCP-Ser were prepared as previously described (74, 82, 94). The preparation of S100A8 is provided as Supplemental Information. Protein stocks were thawed and prepared for use by buffer exchange using Amicon Ultra-0.5 centrifugal spin-filters (MWCO 10 kDa). Three exchanges were performed using ice-cold AMA buffer, centrifuging at 13,000 rpm, 5 min, 4°C. The final protein concentration was calculated from the absorbance at 280 nm (*A*_280_) and the corresponding extinction coefficient (Table S7).

### General methods for bacterial culture

Bacteria from freezer stocks were streaked onto LB plates and incubated overnight at 37°C for 16–20 h. Overnight cultures were prepared by using five single colonies from each plate to inoculate 2 mL of LB in polypropylene culture tubes (VWR), which were incubated for 12–16 h at 37°C, 250 rpm. For experiments involving protein treatment of bacterial cultures, buffer-exchanged protein or the equivalent volume of AMA buffer was added to the culture at the start of incubation.

### Colony-forming unit counting

At each timepoint, 20 µL aliquots of culture were added to a fresh well within a polystyrene 96-well plate containing 180 µL of AMA buffer per well. This suspension was mixed thoroughly and serially diluted (10× each time) until the appropriate dilution was reached, following which 25 µL of the resulting suspension was used to plate a 60 × 15 mm polystyrene petri dish containing either LB agar (monocultures) or selective medium (cocultures*, Pseudomonas* isolation agar for *P. aeruginosa*, Baird-Parker medium for *S. aureus*). The plates were spread uniformly, incubated at 37°C for 18–24 h, and any colonies formed were enumerated.

### Preparation of culture supernatants for HPLC analysis

At each timepoint, 500 µL aliquots of culture suspensions were collected in polypropylene Eppendorf tubes and centrifuged at 4,000 rpm, 7 min, 4°C to remove the majority of cells/debris. A 150 µL aliquot of the clarified supernatant was diluted 1:1 with 150 µL of ice-cold HPLC-grade methanol (Sigma) and stored at −20°C for >1 h to precipitate salt. The samples were then centrifuged at 13,000 rpm, 10 min, 4°C. Centrifugation was repeated on the resulting supernatant to ensure complete removal of particulates, and 200 µL of the final clarified supernatant was used for HPLC analysis.

### High-performance liquid chromatography (HPLC)

HPLC was performed as previously reported (32, 95).

### RNA extraction, workup, and real-time PCR

Cells were harvested for RNA extraction by transferring 500 µL of culture into a new polypropylene tube containing 500 µL of RNAlater (Sigma), mixing thoroughly and incubating at ambient temperature for 5 min. The suspension was then centrifuged at 13,000 rpm, 10 min, 4°C, and lysis of the resulting cell pellet was performed using methodology adapted from a prior report (96). Briefly, cell pellets were resuspended in 100 µL of RNAse-free water and extracted using 100 µL of 25:24:1 phenol/chloroform/isoamyl alcohol (Sigma). The tubes were subjected to six vortex/heat cycles and subsequently centrifuged at 13,000 rpm for 10 min at ambient temperature. A 100 µL aliquot of the upper aqueous phase was carefully transferred into a new tube. RNA cleanup was then performed following the manufacturer’s directions using a Qiagen RNeasy kit, which included an on-column DNase I treatment step. Following elution of RNA from the column (80 μL), a second round of DNase I treatment was performed using NEB DNase I for 2–3h at 37°C (final volume of 100 μL). RNA was precipitated overnight at −20°C by the addition of 300 μL of ice-cold 100% ethanol and then 10 μL of 3 M NaOAc, pH 5.2. RNA was pelleted by centrifugation at 13,000 rpm, 4°C, 30 min, following which the RNA was washed with ethanol and pelleted again. The air-dried RNA pellet was resuspended in nuclease-free H_2_O, and the concentration of extracted RNA was determined by UV absorbance measurements. RNA samples were diluted to 50 ng/µL for subsequent cDNA synthesis. cDNA was prepared using the Protoscript II First Strand cDNA Synthesis Kit (NEB) following the manufacturer’s directions, using 300 ng of RNA template per reaction. Real-time PCR was performed on a Light Cycler 480 II Real-Time PCR System (Roche) at the MIT BioMicroCenter.

## SUPPLEMENTAL MATERIAL

Supplemental Information mbio.03846-24-s0001.pdf Complete experimental methods, supplemental tables, and supplemental figures.

ASM does not own the copyrights to Supplemental Material that may be linked to, or accessed through, an article. The authors have granted ASM a non-exclusive, world-wide license to publish the Supplemental Material files. Please contact the corresponding author directly for reuse.
