# Supplementary material for: Calprotectin-mediated survival of Staphylococcus aureus in coculture with Pseudomonas aeruginosa occurs without nutrient metal sequestration
Source: mBio. 2025 Mar 28;16(5):e03846-24. doi: 10.1128/mbio.03846-24 (PMC12077171; doi:10.1128/mbio.03846-24)
Supplement: Supplemental Information — Complete experimental methods, supplemental tables, and supplemental figures. [file mbio.03846-24-s0001.pdf]

Supplementary Information for

Calprotectin-mediated survival of *Staphylococcus aureus* in coculture with *Pseudomonas aeruginosa* occurs without nutrient metal sequestration

Wei H. Lee[1], Emily M. Zygiel[1], Celis H. Lee,[1] Amanda G. Oglesby[2,3,*], Elizabeth M. Nolan[1,*]

[1]Department of Chemistry, Massachusetts Institute of Technology, Cambridge, MA 02139, United States.

[2]Department of Pharmaceutical Sciences, School of Pharmacy, University of Maryland, Baltimore, MD 21201, United States.

[3]Department of Microbiology and Immunology, School of Medicine, University of Maryland, Baltimore, MD 21201, United States.

*Corresponding authors: lnolan@mit.edu, aoglesby@rx.umaryland.edu

This PDF file includes:

Table of Contents
Experimental Materials and Methods
Tables S1-S16
Supplementary Figures S1-S20
Supplementary References

TABLE OF CONTENTS

Supplementary Experimental

General Materials and Methods.. S4
Instrumentation.. S6
Microbiology.. S7
Metabolite Detection by HPLC... S11
Design of Synthetic Gene for S100A9(H91A)(H95A)... S12

Supplementary Tables

Table S1. Terminology and supplementation used to prepare chemically defined media utilized in this work…………………………………………………………………………………. S13
Table S2. Primers used for real-time PCR of *P. aeruginosa* and *S. aureus*……………………...... S14
Table S3. Nomenclature of human calprotectin variants………………………………………....... S15
Table S4. Primers used for subcloning and site-directed mutagenesis of S100A8....................... S15
Table S5. Primers used for subcloning and site-directed mutagenesis of S100A9....................... S15
Table S6. Templates and primer pairings used in site-directed mutagenesis of CP subunits……. S16
Table S7. Molecular weights and extinction coefficients for proteins used in this work............. S16
Table S8. Bacterial strains used in this study.. S17
Table S9. Standard curves for real-time PCR of genes associated with Fe starvation responses in *P. aeruginosa* and *S. aureus* cocultures.. S17
Table S10. Standard curves for real-time PCR of genes associated with the cell envelopes in monocultures and cocultures of *P. aeruginosa* and *S. aureus* cocultures..................................... S18
Table S11. Mass spectrometric analysis of CP, CP site variants and S100A8.............................. S19
Table S12. Analytical SEC retention volumes and calculated molecular weights of CP and CP site variants in the absence and presence of Ca(II).. S19
Table S13. Representative metal analysis of chemically defined medium……………………… S20
Table S14. Significance testing results for endpoint metabolite levels produced by *P. aeruginosa*.. S20
Table S15. Significance testing results for the effect of CP and metal depletion on coculture growth.. S21
Table S16. Significance testing results for coculture growth experiments.................................. S22

Supplementary Figures

Figure S1. Detection of methylated PCH in supernatants derived from cocultures of *P. aeruginosa* and *S. aureus*... S23
Figure S2. The presence of CP does not significantly affect the relative abundance of methylated PCH detected in cocultures of *P. aeruginosa* and *S. aureus*.. S24
Figure S3. Effect of CP on *P. aeruginosa* PAO1 (A) and *S. aureus* JE2 (B) growth in coculture… S24
Figure S4. Effect of CP on *P. aeruginosa* JSRI-1 (A) and *S. aureus* JE2 (B) growth in coculture... S25
Figure S5. Effect of CP on *P. aeruginosa* PA14 (A) and *S. aureus* Newman (B) growth in coculture……………………………………………………………………………………….. S25
Figure S6. Effect of CP on *P. aeruginosa* PA14 (A) and *S. aureus* COL (B) growth in coculture... S26

Figure S7. Effect of CP on *P. aeruginosa* PA14 (A) and *S. aureus* JE2 (B) growth in coculture in Tris:TSB medium……………………………………………………………………… S26
Figure S8. Effect of CP concentration on *P. aeruginosa* PA14 (A) and *S. aureus* JE2 (B) growth in coculture.. S27
Figure S9. Effect of CP and metal depletion on monocultures of *P. aeruginosa* and *S. aureus*.... S27
Figure S10. The CP protein scaffold does not perturb the expression of genes that affect the cell envelopes of *P. aeruginosa* and *S. aureus* in monoculture……………………………………..... S28
Figure S11. Representative SDS-PAGE of purified CP and CP site variants utilized in this study.. S29
Figure S12. Representative SDS-PAGE of purified S100A8 utilized in this study...................... S29
Figure S13. Analytical size exclusion chromatography of LMW standards and standard curve determination……………………………………………………………………………. S30
Figures S14-S17. Analytical size exclusion chromatography of CP site variants and S100A8 utilized in this study……………………………………………………………………………. S31
Figures S18-S19. Circular dichroism spectra of CP site variants and S100A8 utilized in this study………………………………………………………………………………………… S35
Figure S20. Representative plots used for determination of standard curves for real-time PCR..... S37

Supplementary References... S38

SUPPLEMENTARY EXPERIMENTAL

General Materials and Methods

Solutions and buffers. All chemicals and reagents were purchased from commercial vendors and used as received. All solutions were made using Milli-Q water (18.2 MΩ·cm, Milli-Q Academic system). All buffer and metal stocks were filtered (0.2 μm) before use. All solutions and buffers used for microbiology were prepared using metal-free or trace metals basis reagents.

Metal stocks. Stock solutions of metal salts were prepared by dissolving high purity metal salts (Sigma) in Milli-Q water (18.2 MΩ·cm) using acid-washed volumetric glassware. The stock solutions (Ca, Mg, Mn, Ni, Cu, Zn) were then stored in polypropylene tubes. The following metal salts were used: $CaCl_2 \cdot 2H_2O$ (99.0% BioReagent), $MgSO_4$ (99.99%), $MnCl_2 \cdot 4H_2O$ (99.99%), $NiSO_4 \cdot 6H_2O$ (99.99%), $CuSO_4 \cdot 5H_2O$ (99.999%), anhydrous $ZnCl_2$ (99.999%). Iron stock solutions were prepared immediately before use by dissolving $(NH_4)_2Fe(SO_4)_2 \cdot 6H_2O$ (99.997%) in Milli-Q water under ambient conditions.

Site-directed mutagenesis. To generate the CP variants first reported in this work (**Table S3**), site-directed mutagenesis was performed using a modified QuikChange protocol (Stratagene, currently Agilent) as previously reported (1). The templates and primer pairings for mutagenesis are listed in **Tables S4 – S6**. For all reactions, the PCR conditions used for mutagenesis were: 95 °C for 30 sec (1 x); [95 °C for 30 sec; 55°C for 1 min; 68 °C for 17 min] (25 x); 4 °C hold (1 x).

Protein overexpression, purification, and handling. CP (S100A8/S100A9 heterodimer), CP variants, S100A9(C3S), S100A12 and mCP-Ser were prepared as previously described (1–3). Briefly, protein was prepared by inducing overexpression of BL21(DE3) *Escherichia coli* cells harboring a pET-41a plasmid containing the appropriate gene. For CP, CP-Ser and their variants, overexpression was performed in 4 L culture batches (2 L of each monomer). Proteins were stored in 20 mM HEPES (Sigma, H3375), 100 mM NaCl (VWR, 0241) at pH 8.0 with or without 5 mM DTT depending on whether the protein contains free Cys residues. As CP and its variants (but not CP-Ser) contain a free Cys residue on each S100A8 and S100A9 subunit, 5 mM of dithiothreitol (DTT) (Sigma, D0632) was added to buffers and solutions used for the preparation and storage of CP and CP site variants. Routine yields of CP and ΔHis_3Asp were 80 – 90 mg / 4 L culture, whereas ΔHis_4 and ΔΔ yielded 30 – 40 mg / 4 L culture.

We note that the ΔHis_3Asp, ΔHis_4 and ΔΔ variants used in this study are based on CP, not CP-Ser. These are new variants expressed and purified for the first time. Consequently, we include the site-directed mutagenesis primers and biochemical characterization of these proteins in this Supplementary Information. All three variants showed the expected secondary structure and Ca(II)-dependent self-association to form tetramers (**Figures S14-S17, Table S12)**.

Proteins were thawed and prepared for use by buffer exchange using Amicon Ultra-0.5 centrifugal spin-filters (MWCO 10 kDa, UFC5010BK). Three exchanges were performed using ice-cold 20 mM Tris (VWR, 97061), 100 mM NaCl at pH 7.5 (AMA buffer), centrifuging at 13,000 rpm, 5 min, 4 °C. The concentration of the resulting protein was determined by measuring the sample absorbance at 280 nm (A_{280}). Samples were loaded onto a Take3 microplate (Biotek) and measured on a Synergy HT microplate reader (Biotek). The resulting A_{280} was used together with the extinction coefficient (**Table S7**) to calculate protein concentration.

Purification of S100A8 homodimer. *Escherichia coli* cells harboring a pET-41a plasmid containing the S100A8 gene were grown to saturation in LB containing 50 µg/mL kanamycin by incubating at 37 °C, 175 rpm, 12 – 16 h. The next day, the saturated overnight cultures were used to inoculate 2 L of LB (1:100 dilution) containing 50 µg/mL kanamycin, followed by incubation of the new culture at 37 °C, 175 rpm. Once the cultures had reached OD_{600} of ~0.6, cultures were cooled in an ice bath until the culture temperature reached 18 °C, at which point overexpression was induced by addition of 250 µM IPTG following which cultures were incubated at 18 °C, 175 rpm for 21 h. The cell pellets were collected by centrifugation (3500 rpm, 15 min, 4 °C), following which they were flash frozen in liquid nitrogen and stored at -80 °C. Typical overexpression yields were usually 2.5 – 3.5 g of cell pellet (wet weight) / L of culture. S100A9 was overexpressed as previously described for CP (1, 2). To purify S100A8, a 3:1 mixture of S100A8 and S100A9 (by cell pellet mass) was lysed and the proteins were solubilized and dialyzed following the procedure reported for the purification of CP (1). After dialysis, the crude mixture was centrifuged (14500 rpm, 10 min, 4 °C) to pellet any precipitate, following which the supernatant was filtered using a 0.2 µm PES bottletop filter (Fisher). Next, the S100A8 homodimer was purified using a modified protocol adapted from the purification of CP. The crude protein mixture was first resolved by anion exchange chromatography on a Capto HiRes Q 10/100 column (Cytiva) using a gradient of 0-20% B (A: 20 mM HEPES, 5 mM DTT, pH 8.0; B: 20 mM HEPES, 1 M NaCl, 5 mM DTT, pH 8) over 15 CV. This yielded two distinct and well-resolved peaks, with the earlier peak (7.5 – 10% B) corresponding to S100A8 and the later peak (~12 – 14% B) corresponding to CP. S100A8 was further processed following the

workflow used to prepare CP; typical yields for S100A8 were around 15-20 mg / 5 L culture (3:1 ratio of S100A8:S100A9 overexpression pellet mass).

Instrumentation

High performance liquid chromatography (HPLC). HPLC was used for the analysis of pseudomonal metabolites as previously reported (4, 5).

Quadrupole time-of-flight mass spectrometry (QTOF-MS). QTOF-MS was used for analysis of CP, CP site variants, and S100A8 **(Table S11)**. QTOF-MS was performed on an Agilent Infinity 1260 LC platform equipped with a 6230 TOF system utilizing an Agilent Jetstream ESI source, housed within the MIT Department of Chemistry Instrumentation Facility. A 3-5 μL aliquot of sample was injected for analysis. The solvent system used was H_2O/0.1% formic acid (solvent A) and MeCN/0.1% formic acid (solvent B), and the instrument was run in positive polarity mode, at a flow rate of 0.3 mL/min. Protein samples (0.2-1 mM) were buffer exchanged four times against 10 mM sodium acetate to remove buffer components. The samples were then diluted in an equal volume of 10 mM sodium acetate, filtered using a 0.2 μm filter, and loaded into clear glass vials fitted with a 200 μL glass insert (Agilent). Samples were run on a Poroshell 300SB-C18 column (Agilent, 660750-902) with the following gradient: 0-2 min: 0 %B, 2-12 min: 2-90 %B, 12-14 min: 90 %B, 14-16 min: 90-0 %B. The resulting mass spectra was deconvoluted using MassHunter Bioconfirm software (Agilent).

Inductively coupled plasma-mass spectrometry (ICP-MS). ICP-MS was used for metal analysis of CDM. Samples were analyzed on an Agilent 7900 ICP-MS instrument (MIT CEHS Bioimaging and Chemical Analysis core facility) to measure the concentrations of metals present. Cell suspensions were added to 5% HNO_3 (ARISTAR Ultra, Ultrapure for Analysis, VWR) in polypropylene 5 mL tubes (MTC Bio, C2540) and supplemented with 1 ppb Tb (terbium) internal standard (Agilent, 5190-8590). After microwave digestion, samples were transferred to clear polypropylene sample cups (VWR, 15070-089) and loaded onto the autosampler. The instrument was run in He gas mode and tuned according to standard facility protocol before each use. Each batch of samples included a series of known standards to obtain standard curves for instrument calibration. The standards consisted of five 10X serial dilutions of Environmental Calibration Standard (Agilent 5183-4688) in 5% HNO_3 (starting from a 1:10 dilution from neat), and an additional 5% HNO_3 blank.

Analytical size exclusion chromatography. Analytical size exclusion chromatography (SEC) was used for biophysical characterization of CP site variants and S100A8 (**Table S12**). Methodology for analytical size exclusion chromatography was adapted from a reported protocol (1). Briefly, analytical SEC was performed on an ÄKTA purifier (GE Healthcare Life Sciences) equipped with a Superdex 75 10/300 GL column (GE Healthcare Life Sciences) and fitted with a 100 µL sample loop. Experiments were conducted at 4 °C, with the column pre-equilibrated with at least one column volume of running buffer prior to each experiment. For each run, 300 µL of 120 µM protein was injected into the sample loop and the loop was then emptied using 500 µL of running buffer. Samples were then eluted over one column volume using a flow rate of 0.35 mL/min. Calibration was performed using the LMW Gel Filtration Calibration Kit (Cytiva). Standard samples were prepared following manufacturer instructions, noting that carbonic anhydrase was dissolved in water as it precipitated when dissolved in the recommended buffer (1). A 300 μL aliquot of each standard (of the appropriate concentration) was used for injection. The elution times of the standards were then used to calculate the partition coefficient based on their known masses, generating a standard curve (1). Finally, the elution times of the assayed proteins were used to determine the apparent molecular weight of each protein sample.

Circular dichroism (CD) spectroscopy. CD spectroscopy was used for the biophysical characterization of CP variants and S100A8 (**Figures S18 and S19**). CD spectra were collected using a JASCO J-1500 spectrometer set to 25 °C housed in the MIT Biophysical Instrumentation Facility. Samples were collected using a quartz CD cell (Hellma, 1 mm path length), with a measurement window of 260 – 195 nm (1 nm interval, 3 sec averaging times). Protein samples were prepared for CD by diluting concentrated protein stock solutions into buffer (1 mM Tris-HCl, 500 mM EDTA, pH 8.5) for a final concentration of 10 µM (300 µL) (6). To examine how Ca(II) affects protein folding and structure, Ca(II) was added (from a 100 mM stock) for a final concentration of 2 mM and the sample was incubated at room temperature for 3 min. The averaged spectral data from three independent scans is reported.

Microbiology

Growth media. All growth media with the exception of chemically defined medium (CDM) was purchased from commercial vendors. Luria-Bertani (LB, Miller) medium (BD Difco), Tryptic Soy Broth (TSB) (BD Difco), *Pseudomonas* Isolation Agar (Sigma, BD Difco) and Baird-Parker medium (Sigma, BD Difco) were dissolved into Milli-Q water, sterilized and prepared according to manufacturer recommendations. Sterilized Baird-Parker medium was completed by addition of egg yolk tellurite emulsion (Sigma, BD

Difco). Tris:TSB medium was prepared by mixing sterilized antimicrobial activity (AMA) buffer (20 mM Tris, 100 mM NaCl, pH 7.5) and TSB in a 62:38 ratio, and filtering (0.2 μm) the resulting medium under sterile conditions. Tris:TSB medium was supplemented with 2 mM of Ca(II) immediately before use.

Chemically-defined medium (CDM). Unsupplemented CDM (dCDM) was prepared as previously described (7). Metal-replete CDM was prepared by supplementing dCDM with the following metals immediately prior to use: 1 mM Ca(II), 0.3 μM Mn(II), 5 μM Fe(II), 0.1 μM Ni(II), 0.1 μM Cu(II) and 6 μM Zn(II). CDM depleted of one metal (e.g. Mn-depleted, Fe-depleted and Zn-depleted CDM) was prepared by omitting the corresponding metal from the above supplementation of dCDM, and metal-depleted CDM was prepared by omitting Mn, Fe and Zn from the above supplementation of dCDM (**Table S1**).

General methods for bacterial culture. The full list of bacterial strains used in this study is listed in **Table S8**. Bacteria from freezer stocks were streaked onto LB plates and incubated overnight at 37 °C for 16 – 20 h. Overnight cultures were prepared by using five single colonies from each plate to inoculate 2 mL of LB in 14 mL polypropylene culture tubes (VWR, 60818-703), which were then incubated at 37 °C for 12-16 h, at 250 rpm. For experiments involving treatment of bacterial cultures with protein, 20 μM of the buffer exchanged protein or the equivalent volume of AMA buffer was added to the culture at the start of incubation. To prepare Zn(II)-bound CP, 20 μM of CP was added into 2 ml of metal-depleted CDM and incubated at ambient temperature for 3 min. Subsequently, Zn(II) was added at 2:1 molar ratio to CP (equimolar to available Zn(II)-binding sites), followed by gentle mixing and incubation at ambient temperature for 5 min. We note that we observed that addition of Zn(II) to CP at protein concentrations of 80 μM and above led to the formation of a cloudy precipitate after incubation at ambient temperature.

Unless otherwise specified, monocultures of *P. aeruginosa* and *S. aureus* cultures were diluted from overnight starter cultures into sterile media using the following starting OD_{600} values: *P. aeruginosa*: 0.005 (log (CFU/mL) = 6.8 – 7.0), *S. aureus*: 0.02 (log (CFU/mL) = 7.2 – 7.4). For cocultures of *P. aeruginosa* and *S. aureus*, cultures were diluted from overnight starter cultures into sterile media using the same starting OD_{600} values.

Culture turbidity measurements. Culture turbidity was determined by measuring the absorbance at 600 nm (OD_{600}) using a Beckman Coulter DU800 spectrophotometer. A 1 mL aliquot of undiluted or diluted

culture was transferred into disposable polystyrene cuvettes (VWR, 97000-584) for sample measurement at ambient temperature.

Colony-forming unit counting. At each timepoint, 20 μL aliquots of culture were added to added to a fresh well within a polystyrene 96-well plate (Corning, 3595) containing 180 μL of AMA buffer per well. This suspension was mixed thoroughly and serially diluted (10X each time) until the appropriate dilution was reached, following which 25 μL of the suspension was used to plate a 60 x 15 mm polystyrene petri dish (VWR, 25384-092, or Fisher Scientific, FB0875713A) containing either LB agar (monocultures) or selective medium (cocultures, *Pseudomonas* isolation agar for *P. aeruginosa*, Baird-Parker medium for *S. aureus*). The plates were spread uniformly and incubated at 37 °C for 18 – 24 h, following which any formed colonies were enumerated.

RNA extraction. Cells were harvested for RNA extraction by transferring 500 μL of culture into a new polypropylene tube containing 500 μL of RNAlater® (Sigma, R0901), mixing thoroughly and incubating at ambient temperature for 5 min. The suspension was then centrifuged at 13,000 rpm, 10 min, 4 °C and the supernatant was discarded. The resulting cell pellet was either used immediately, or was snap frozen in liquid N_2 and stored at -80 °C until use. Cell lysis was performed using methodology adapted from a prior report (8). Cell pellets were resuspended in 100 μL of water inside an Eppendorf tube. The tube was vigorously vortexed (3000 rpm) for 3 min, after which 100 μL of 25:24:1 phenol/chloroform/isoamyl alcohol (Sigma, 77617) was added. The tubes were subjected to six vortex/heat cycles, each consisting of a one-minute vortex at 3000 rpm, followed immediately by a five-minute incubation at 70 °C. Next, the tube was centrifuged at 12,000 g for 10 min at ambient temperature. A 100 μL aliquot of the upper aqueous phase was carefully removed and transferred into a new tube. A 700 μL aliquot of RLT lysis buffer (Qiagen RNeasy kit) was then added. The RNA extraction protocol continued according to manufacturer directions with the QIAgen RNAeasy kit, noting to include an on-column DNaseI treatment step using 30 μL of DNaseI + 80 μL of buffer PDD (RNase-free DNase set, Qiagen) for 1-2 h at ambient conditions. Following elution of RNA (80 μL) from the column, a second round of DNaseI treatment was performed using 10 μL of NEB DNaseI (New England Biosciences) + 10 μL of 10X reaction buffer, for 2 – 3 h at 37 °C (final volume of 100 μL).

RNA workup. After extraction, RNA was then precipitated overnight at -20 °C by the addition of 300 μL of ice-cold 100% ethanol and 10 μL of 3M NaOAc pH 5.2 (Thermo Scientific, R1181). The samples were precipitated by centrifugation at 14,000 rpm, 4 °C, 30 min. The RNA was resuspended and washed once

more by careful removal of supernatant, resuspension in 1 mL of ice-cold ethanol and centrifuging at 14,000 rpm, 4 °C, 20 min. After the final spin, the supernatant was removed and the tube containing the RNA pellet was inverted to dry for 5 min under ambient conditions. Lastly, the RNA pellet was resuspended in 30 µL of nuclease-free H_2O (VWR, E476) and the concentration of extracted RNA was determined by UV measurements using a Take3 microplate (Biotek) and a Synergy HT microplate reader (Biotek). Typical RNA yields were around 90-450 ng/µL. Aliquots of each RNA sample were transferred into a new tube, diluted to 50 ng/µL for subsequent cDNA synthesis, and stored at -20 °C. RNA stocks were snap frozen in liquid N_2 and stored at -80 °C (thawed only once). 1 µL of the diluted RNA was added to 199 µL of nuclease-free H_2O and stored at 4 °C as the negative control for subsequent RT-PCR (no reverse transcription).

cDNA synthesis and real-time PCR. cDNA was prepared using the Protoscript II First Strand cDNA Synthesis Kit (NEB) according to manufacturer directions, using 300 ng of input RNA template per reaction for targets associated with Fe starvation responses and 100 ng of input RNA template per reaction for targets associated with modifications to the cell envelopes. The following temperature cycle was used: 42 °C (1 hour) → 80 °C (5 min) → 4 °C (hold). cDNA was stored at 4 °C after synthesis. qPCR reactions were loaded into a clear 96-well plate (VWR, 82006-678) from master-mixes prepared in polypropylene tubes. Per reaction, 3 µL of diluted cDNA (1:10), 5 µL of PowerUp SYBR Green Master Mix (ThermoFisher, A25776), 1 µL of 10 µM forward primer and 1 µL of 10 µM reverse primer (final 1 µM each) was added. The plates were sealed with transparent film (VWR, 60941-072), briefly centrifuged and run on a Light Cycler 480 II Real-Time PCR System (Roche) at the MIT BioMicroCenter using the following cycler conditions: i) pre-denaturation: 95 °C (30 sec), followed by ii) [95 °C (10 sec) → 60 °C (10 sec) → 72 °C (30 sec)] x 40 cycles, with detection mode set to SYBR Green. C_T values were determined using the default software preset set to absolute quantification, with second-derivative fitting using the default threshold value. To identify the effective dynamic range for each assayed gene, cDNA synthesis reactions were performed using 0.1, 1, 10, 100, 300 and 600 ng of RNA extracted from cocultures of *P. aeruginosa* and *S. aureus* treated with or without CP. The resulting cDNA samples were then used for real-time PCR and the gene-wise C_T values obtained were subsequently plotted against the log of the effective dilution factor (representative example in **Figure S20**). The effective dynamic range for each gene was identified using linear fitting and problematic datapoints were omitted (**Tables S9 and S10**). The standard curves revealed the appropriate amount of input RNA for cDNA synthesis in order to achieve real-time PCR detection within the effective dynamic range across all assayed genes.

Metabolite Detection by HPLC

General methods. For each analyzed metabolite, standard curves (for quantification) were prepared by dissolving commercially available or purified compounds in 1:1 H_2O/0.1% trifluoroacetic acid and MeCN/0.1% trifluoroacetic acid in preparation for HPLC. Pyocyanin (PYO) and phenazine-1-carboxylic acid (PCA) were obtained from Sigma. Pyoverdine (PVD) was extracted from *P. aeruginosa* PAO1 supernatants and purified as previously reported (5). Pyochelin (PCH) was extracted and purified using a modified protocol adapted from previous reports (4, 5, 9, 10).

Preparation of culture supernatants for HPLC analysis. At each timepoint, 500 µL aliquots of culture suspensions were collected in polypropylene Eppendorf tubes and centrifuged at 4,000 rpm, 7 min, 4 °C to remove the majority of cells/debris. The clarified supernatant was transferred into a new tube and stored at -20 °C. Supernatants were thawed on ice and a 150 µL aliquot of supernatant was diluted 1:1 with 150 µL of ice-cold HPLC-grade methanol (Sigma, 34860-1L-R) for a final volume of 300 µL. The diluted samples were stored at -20 °C for >1 h to precipitate salt, following which they were centrifuged at 13,000 rpm, 10 min, 4 °C to pellet any particles. 250 µL of the supernatant was carefully transferred into a new ice-cold tube and centrifuged again at 13,000 rpm, 10 min, 4 °C to ensure complete removal of any particulates. 200 µL of the supernatant was transferred into an HPLC vial fitted with a glass insert and kept on ice until analysis.

Design of Synthetic Gene for S100A9(H91A)(H95A)

The synthetic gene for human S100A9(H91A)(H95A) was designed with terminal cut sites for NdeI and XhoI (underlined) for subsequent insertion into a pET-41a vector. The synthetic gene was ordered from Azenta (Genewiz) and optimized for expression in *E. coli*. In the nucleotide and amino acid sequences below, the start codon and the corresponding Met residue is highlighted in bold:

***E. coli* optimized nucleotide sequence for S100A9(H91A)(H95A):**

CAT**ATG**ACCTGCAAAATGAGTCAGCTGGAACGCAACATTGAAACCATTATTAACACCTTTCA
TCAGTATAGCGTGAAACTGGGCCATCCGGATACCCTGAACCAAGGCGAATTTAAAGAACTG
GTGCGCAAAGATCTGCAGAACTTTCTGAAAAAAGAAAACAAAAACGAAAAAGTGATTGAAC
ATATTATGGAAGATCTGGATACCAACGCGGATAAACAGCTGAGCTTTGAAGAATTTATTATG
CTGATGGCGCGCCTGACCTGGGCGAGCGCGGAAAAAATGGCGGAAGGCGATGAAGGCCCG
GGCCATCATCATAAACCGGGCCTGGGCGAAGGCACCCCGTAACTCGAG

Translated sequence for S100A9(H91A)(H95A):

H **M** T C K M S Q L E R N I E T I I N T F H Q Y S V K L G H P D T L N Q G E F K E L V R K D L Q
N F L K K E N K N E K V I E H I M E D L D T N A D K Q L S F E E F I M L M A R L T W A S A E
K M A E G D E G P G H H H K P G L G E G T P

SUPPLEMENTARY TABLES

Table S1. Terminology and supplementation used to prepare chemically defined media (CDM) utilized in this work.

Medium	Ca (1 mM)	Mn (0.3 µM)	Fe (5 µM)	Zn (6 µM)	Ni (0.1 µM)	Cu (0.1 µM)
Unsupplemented CDM (dCDM)	×	×	×	×	×	×
Metal-replete CDM	✓	✓	✓	✓	✓	✓
Mn-depleted CDM	✓	×	✓	✓	✓	✓
Fe-depleted CDM	✓	✓	×	✓	✓	✓
Zn-depleted CDM	✓	✓	✓	×	✓	✓
Metal-depleted CDM	✓	×	×	×	✓	✓

Table S2. Primers used for real-time PCR of *P. aeruginosa* and *S. aureus*.

Primer	Sequence (5' to 3')	Reference
Pseudomonas aeruginosa		
16S.For	GGAGAAAGTGGGGGATCTTC	(11)
16S.Rev	CCGGTGCTTATTCTGTTGGT	(11)
pvdS.For	CAGGCGCTCGAACAGAAATA	-
pvdS.Rev	TCGCGGATCATGAAGTTGAC	-
feoB.For	CGAGAGGAACAGGTACATCAAG	-
feoB.Rev	GATGTTCTTCTTCGCCATCAAC	-
prrF.For	AACTGGTCGCGAGATCAGC	(12)
prrF.Rev	CCGTGATTAGCCTGATGAGGAG	(12)
hasR.For	CGTGGCGTCGAGTACCAG	(13)
hasR.Rev	GGTCTTCGAACAGAAGTCGTTG	(13)
speE2.For	CTCTCTATACCCGCCAGTTCTA	-
speE2.Rev	GGACCTGGGAGAAGACACT	-
arnT.For	TTCTTCTGGCACGAACACAT	(14)
arnT.Rev	CAGCAACGGCAGGTAGAA	(14)
PA3432.For	GCGGAGATGTTGCTGTTCT	-
PA3432.Rev	AGCATGATCACCAGGAGGAT	-
Staphylococcus aureus		
sigA.For	TGGTGCTGGATCTCGACCTA	(13)
sigB.Rev	TGCAATTGCTGACCAAGCAC	(13)
cntA.For	CTGCGTCAGCATCAAATGGC	-
cntA.Rev	TCCGCATGTTTACGGTGGAT	-
sirA.For	TCCGCCAGCTAAGTTCCAAG	-
sirA.Rev	TCCAAATGCGAAAGATGCTGC	-
sbnC.For	GCAATACGGCGCATTACCAG	-
sbnC.Rev	AGCTGATGTGCTGTTAGCCA	-
isdC.For	CTGCCAAAGATGAACGCACT	(13)
isdC.Rev	GCACCTGCTACATCAGTTGGT	(13)
cap8F.For	GAGAGTATGCCGAAGAGTATGG	-
cap8F.Rev	CTTACACCACTTGCCGAATAAAT	-
comK.For	TGCAAGAGCGTTCAATGATTT	-
comK.Rev	CGACCAGCATTTGATGATGAC	-
fosB.For	TTTGAGCTTGCAGGCCTAT	-
fosB.Rev	GCCAATATTTAAATTCGCTGTCATC	-

Table S3. Nomenclature of human calprotectin variants.

Protein	S100A8 amino acid substitutions(s)	S100A9 amino acid substitutions(s)	Description	Ref.
Calprotectin (CP)	N/A	N/A	Wild-type CP	This work
CP ΔHis$_3$Asp	H83A, H87A	H20A, D30A	Functional His$_6$ site	This work
CP ΔHis$_4$	H17A, H27A	H91A, H95A	Functional His$_3$Asp site	This work
CP ΔΔ	H17A, H27A, H83A, H87A	H20A, D30A, H91A, H95A	No functional transition metal-binding sites	This work
CP-Ser	C42S	C3S	Cys → Ser variant	(1)
CP-Ser Δ101	C42S	C3S, G102Stop	Truncated S100A9 tail	(2)
CP-Ser-AAA	C42S	C3S, H103A, H104A, H105A	Tail variant with decreased contribution to the His$_6$ site	(2)

Table S4. Primers used for subcloning and site-directed mutagenesis of S100A8 (1).[a]

Primer	Sequence (5' to 3')
H17A-1	CGACGTTTAC<u>GCG</u>AAATACAGCCTG
H17A-2	GCTGCAAATG<u>CGC</u>TTTATGTCGGAC
H27A-1	CAAGGGTAACTTT<u>GCG</u>GCGGTCTATCGTG
H28A-2	GTTCCCATTGAAA<u>CGC</u>CGCCAGATAGCAC
H83A-1	CAAAATGGGTGTTGCGGCC<u>GCG</u>AAGAAGAGCCACGAAG
H83A-2	GTTTTACCCACAACGCCGG<u>CGC</u>TTCTTCTCGGTGCTTC
**(H83A)-H87A-1**	GCC*GCG*AAGAAGAGC<u>GCG</u>GAAGAGAGCCATAAAG
**(H83A)-H87A-2**	CGG*CGC*TTCTTCTCG<u>CGC</u>CTTCTCTCGGTATTTC

[a] Mutations already present in the template (see **Table S6**) are listed in parentheses and their corresponding codons are italicized and highlighted in blue. Codons containing the mutations are underlined and highlighted in red.

Table S5. Primers used for subcloning and site-directed mutagenesis of S100A9 (1).[a]

Primer	Sequence (5' to 3')
H20A-1	CATTATCAATACCTTC<u>GCG</u>CAATACTCCGTCAAATTG
H20A-2	GTAATAGTTATGGAAG<u>CGC</u>GTTATGAGGCAGTTTAAC
D30A-1	CAAATTGGGTCATCCG<u>GCG</u>ACGCTGAACCAGGGCGAG
D30A-2	GTTTAACCCAGTAGGC<u>CGC</u>TGCGACTTGGTCCCGCTC
H91A-1	GTCTGACGTGGGCGAGC<u>GCG</u>GAGAAAATGCATGAAGG
H91A-2	CAGACTGCACCCGCTCG<u>CGC</u>CTCTTTTACGTACTTCC
**(H91A)-H95A-1**	GAGC*GCG*GAGAAAATG<u>GCG</u>GAAGGTGATGAAGGTCC
(H91A)-H95A-2	CTCG*CGC*CTCTTTTAC<u>CGC</u>CTTCCACTACTTCCAGG

[a] Mutations already present in the template (see **Table S6**) are listed in parentheses and their corresponding codons are italicized and highlighted in blue. Codons containing the mutations are underlined and highlighted in red.

Table S6. Templates and primer pairings used in site-directed mutagenesis of CP subunits (1).

Template	Product	Primer Pairing
pET-41a-S100A8	pET-41a-S100A8(H17A)	H17A-1, H17A-2
pET-41a-S100A8	pET-41a-S100A8(H83A)	H83A-1, H83A-2
pET-41a-S100A8(H17A)	pET-41a-S100A8(H17A)(H27A)	H27A-1, H27A-2
pET-41a-S100A8(H83A)	pET-41a-S100A8(H83A)(H87A)	(H83A)-H87A-1, (H83A)-H87A-2
pET-41a-S100A8(H17A)(H27A)	pET-41a-S100A8(H17A)(H27A)(H83A)	H83A-1, H83A-2
pET-41a-S100A8(H17A)(H27A)(H83A)	pET-41a-S100A8(H17A)(H27A)(H83A)(H87A)	(H83A)-H87A-1, (H83A)-H87A-2
pET-41a-S100A9	pET-41a-S100A9(H20A)	H20A-1, H20A-2
pET-41a-S100A9	pET-41a-S100A9(H91A)	H91A-1, H91A-2
pET-41a-S100A9(H20A)	pET-41a-S100A9(H20A)(D30A)	D30A-1,
pET-41a-S100A9(H20A)(D30A)	pET-41a-S100A9(H20A)(D30A)(H91A)	H91A-1, H91A-2
pET-41a-S100A9(H20A)(D30A)(H91A)	pET-41a-S100A9(H20A)(D30A)(H91A)(H95A)	(H91A)-H95A-1, (H91A)-H95A-2

Table S7. Molecular weights and extinction coefficients for proteins used in this work.[a]

Protein	Molecular weight (g/mol)	ε_{280} ($M^{-1}cm^{-1}$)	Reference
Calprotectin (CP)	24076.5	18450	(1)
CP ΔHis_3Asp	23834.3	18450	This work
CP ΔHis_4	23812.3	18450	This work
CP ΔΔ	23570.1	18450	This work
CP-Ser	24044.3	18450	(1)
CP-Ser AAA	23846.1	18450	(2)
CP-Ser-Δ101	22738.9	18450	(2)
S100A8 (homodimer)	21669.0	22920	This work
S100A9(C3S) (homodimer)	26451.8	13980	(2)
S100A12 (homodimer)	21150.0	5960	(2)
Murine CP-Ser	23279.2	5960	(3)

[a] Molecular weights and extinction coefficients at 280 nm were calculated using the Expasy ProtParam tool (web.expasy.org/protparam/)

Table S8. Bacterial strains used in this study.

Strain	Description	Reference
***Pseudomonas aeruginosa* PAO1**	Laboratory strain	(15)
***Pseudomonas aeruginosa* PA14**	Clinical isolate UCBPP-PA14 (courtesy of Dianne Newman)	(16)
***Pseudomonas aeruginosa* JSRI-1**	Clinical isolate obtained from the lungs of 8-year-old CF patient	(17)
***Staphylococcus aureus* USA300 JE2**	MRSA strain USA300 cured of three plasmids, Tet^S, Ery^S	NTML (18) (NARSA)
***Staphylococcus aureus* Newman**	Antibiotic-susceptible strain isolated from human infection (courtesy of Mark Smeltzer)	(19)
***Staphylococcus aureus* COL**	Methicillin-resistant clinical isolate (courtesy of Mark Smeltzer)	(20)

Table S9. Standard curves for real-time PCR of genes associated with Fe starvation responses in *P. aeruginosa* and *S. aureus* cocultures.

**Gene**	**Conc. Range (-CP) (ng/µL)**	**R^2 value**	**Conc. Range (+CP) (ng/µL)**	**R^2 value**
16S	10–300	0.9827	10–600	0.9981
pvdS	10–300	0.9937	10–300	0.9882
feoB	1–300	0.9836	10–300	0.9939
prrF	10–300	0.9892	10–600	0.9866
hasR	100–600	0.9955	10–300	0.9908
sigA	10–300	0.9962	10–300	0.9998
cntA	10–300	0.9946	10–600	0.9905
sirA	10–600	0.9999	0.1–300	0.9874
sbnC	10–300	0.9996	0.1–300	0.9867
isdC	10–300	0.9895	10–600	0.9967

Table S10. Standard curves for real-time PCR of genes associated with the cell envelopes in monocultures and cocultures of *P. aeruginosa* and *S. aureus*.

**Monoculture/ Gene**	**Conc. Range (untreated) (ng/μL)**	**R^2 value**	**Conc. Range (+CP) (ng/μL)**	**R^2 value**	**Conc. Range (+ΔΔ) (ng/μL)**	**R^2 value**
16S	1–100	0.9863	0.1–300	0.9947	1–300	0.9871
speE2	1–300	0.9896	10–300	0.9974	1–300	0.9840
arnT	1–300	0.9931	10–300	0.9995	1–300	0.9909
PA3432	1–100	0.9907	1–100	0.9926	10–300	0.9827
sigA	1–100	0.9965	1–100	0.9900	10–100	0.9899
cap8F	1–300	0.9953	1–100	0.9998	1–100	0.9983
comK	10–300	0.9998	1–100	0.9994	10–300	0.9983
fosB	10–300	0.9996	1–300	0.9911	10–300	0.9978
**Coculture/ Gene**	**Conc. Range (untreated) (ng/μL)**	**R^2 value**	**Conc. Range (+CP) (ng/μL)**	**R^2 value**	**Conc. Range (+ΔΔ) (ng/μL)**	**R^2 value**
16S	10–300	0.9827	10–600	0.9981	1–100	0.9958
speE2	10–300	0.9937	10–300	0.9882	1–100	0.9946
arnT	10–300	0.9933	1–300	0.9865	10–300	0.9998
PA3432	10–300	0.9939	0.1–100	0.9933	1–100	0.9928
sigA	100–600	0.9955	10–300	0.9908	10–300	0.9860
cap8F	10–300	0.9970	1–100	0.9999	10–300	0.9869
comK	10–300	0.9892	0.1–100	0.9892	10–300	0.9971
fosB	10–300	0.9985	0.1–100	0.9886	10–300	0.9886

Table S11. Mass spectrometric analysis of CP, CP site variants and S100A8.[a]

Protein	S100A8 calculated mass (g/mol)	S100A8 observed mass (g/mol)	S100A9 calculated mass ± ^{N}Met (g/mol)[b]	S100A9 observed mass (g/mol)
Calprotectin (CP)	10834.5	10834.4	13242.0	n.d.[c]
			13110.8	13110.6
CP ΔHis$_3$Asp	10702.4	10702.3	13131.9	n.d.[c]
			13000.7	13000.6
CP ΔHis$_4$	10702.4	10702.3	13109.9	n.d.[c]
			12978.7	12978.6
CP ΔΔ	10570.3	10570.2	12999.8	n.d.[c]
			12868.6	12868.6
S100A8 (heterodimer)	10834.5 (monomer)	10834.6		

[a] Molecular weights were calculated using the Expasy ProtParam tool (web.expasy.org/protparam/). [b] The N-terminal methionine residue of S100A9 is sometimes cleaved during overexpression in *E. coli*; masses are shown for both the full-length and shortened forms of the S100A9 subunit. [c] Mass not found during deconvolution of the obtained raw data.

Table S12. Analytical SEC retention volumes and calculated molecular weights of CP and CP site variants in the absence and presence of Ca(II).[a]

Protein	Retention volume −Ca(II) (mL)	Calculated molecular weight (kDa)[b]	Retention volume +Ca(II) (mL)	Calculated molecular weight (kDa)[b]
Calprotectin (CP)	11.1	36.3	10.3	50.4
CP ΔHis$_3$Asp	11.0	37.9	10.3	50.4
CP ΔHis$_4$	11.5	30.8	10.5	46.5
CP ΔΔ	11.0	37.9	10.2	52.5
S100A8 (heterodimer)	11.8	27.0	10.7<br>11.8	43.2<br>27.7

[a] Protein (120 µM) was injected and eluted over a Superdex 75 10/300 GL column (GE Healthcare Life Sciences) in 20 mM HEPES, 150 mM NaCl, pH 8 using a 0.35 mL/min flow rate. [b] Molecular weights were calculated using a calibration curve spanning a range of 6.5 to 75 kDa.

Table S13. Representative metal analysis of chemically defined medium (CDM).

	Unsupplemented CDM		Metal-replete CDM	
**Element**	**ppb**	**µM**	**ppb**	**µM**
**Mg**	37397.6	1538.6	25733.6	1058.8
**Ca**	1370.4	34.19	49230.0	1228.4
**Mn**	< 0.2	< 0.00364	18.2	0.331
**Fe**	< 0.2	< 0.00358	323.1	5.785
**Co**	< 0.2	< 0.00339	0.291	0.00494
**Ni**	< 0.2	< 0.00341	6.80	0.116
**Cu**	< 0.2	< 0.00315	8.20	0.129
**Zn**	10.3	0.158	367.9	5.628

Table S14. Significance testing results for endpoint metabolite levels produced by *P. aeruginosa*.[a]

Figure	Condition	p-value	Comments
2A	−Zn	N.A.	Not detected in both -Zn and metal-replete
2A	−Fe	0.00718*	-
2A	+CP	0.251	-
2B	−Zn	0.0159*	-
2B	−Fe	2.33e-05*	-
2B	+CP	0.0741*	-
3A	−Zn	0.0325*	-
3A	−Fe	0.0323*	-
3A	+CP	0.559	-
3B	−Zn	0.169	-
3B	−Fe	1.52e-04*	-
3B	+CP	0.00238*	-
4A	−Zn	0.0896	-
4A	−Fe	0.00781*	-
4A	+CP	0.00331*	-
4B	−Zn	0.0424*	-
4B	−Fe	0.00306*	-
4B	+CP	1.18e-04*	-
4C	−Zn	0.162	-
4C	−Fe	0.0308*	-
4C	+CP	0.0301*	-
4D	−Zn	0.0217*	-
4D	−Fe	0.00301*	-
4D	+CP	0.00397*	-

[a]All significance testing at the endpoint (14 h) was performed with respect to the metal-replete condition using a two-sample unpaired student's t-test. Instances where the p-value met the significance threshold of $p < 0.05$ are marked with an asterisk (*).

Table S15. Significance testing results for the effect of CP and metal depletion on coculture growth.[a]

Figure	Condition	Time (h)	p-value	Time (h)	p-value
5A	−Mn	10	0.384	12	0.166
5A	−Mn	14	0.765	16	0.117
5A	−Zn	10	0.720	12	0.581
5A	−Zn	14	0.957	16	0.508
5A	−Fe	10	0.187	12	0.318
5A	−Fe	14	0.354	16	0.818
5A	−Mn, Fe, Zn	10	0.400	12	0.648
5A	−Mn, Fe, Zn	14	0.117	16	0.412
5A	+CP	10	0.00778*	12	0.0339*
5A	+CP	14	0.0515	16	0.100
5A	−Mn, Fe, Zn +CP	10	0.00128*	12	0.0101*
5A	−Mn, Fe, Zn +CP	14	0.0661	16	0.00188*
5B	−Mn	10	0.562	12	0.734
5B	−Mn	14	0.927	16	0.136
5B	−Zn	10	0.482	12	0.327
5B	−Zn	14	0.0676	16	0.175
5B	−Fe	10	9.83e-05*	12	0.882
5B	−Fe	14	0.927	16	0.517
5B	−Mn, Fe, Zn	10	1.55e-05*	12	0.340
5B	−Mn, Fe, Zn	14	0.195	16	0.517
5B	+CP	10	0.159	12	0.0165*
5B	+CP	14	0.0152*	16	0.135
5B	−Mn, Fe, Zn +CP	10	0.143	12	3.55e-04*
5B	−Mn, Fe, Zn +CP	14	4.12e-07*	16	3.55e-05*

[a]All significance testing at the endpoint (14 h) was performed with respect to the metal-replete condition using a two-sample unpaired student's t-test. Instances where the p-value met the significance threshold of $p < 0.05$ are marked with an asterisk (*).

Table S16. Significance testing results for coculture growth experiments

Figure	Condition	Time (h)	p-value	Time (h)	p-value	Time (h)	p-value
6A	+CP	10	0.00540*	12	0.492	14	0.00189*
6A	+ΔHis$_3$Asp	10	0.00687*	12	0.789	14	0.00495*
6A	+ΔHis$_4$	10	7.94e-05*	12	0.00956*	14	0.00305*
6A	+ΔΔ	10	0.00222*	12	0.159	14	0.727
6B	+CP	10	1.73e-04*	12	7.45e-04*	14	0.0312*
6B	+ΔHis$_3$Asp	10	2.06e-04*	12	0.00667*	14	0.0725
6B	+ΔHis$_4$	10	1.38e-04*	12	6.97e-05*	14	0.00416*
6B	+ΔΔ	10	1.51e-04*	12	0.00126*	14	0.367
7A	+CP	10	0.447	12	0.605	14	0.0252*
7A	+Zn(II)	10	0.144	12	0.892	14	0.00332*
7A	+Zn(II)-CP	10	0.384	12	0.336	14	0.00400*
7B	+CP	10	0.00446*	12	0.103	14	1.00
7B	+Zn(II)	10	0.734	12	0.361	14	0.415
7B	+Zn(II)-CP	10	1.64e-05*	12	0.00388*	14	0.00943*
9A	+CP	10	0.272	12	0.0931	14	0.852
9A	+CP-Ser	10	0.141	12	0.113	14	0.708
9A	+Δ101	10	0.739	12	0.0918	14	0.371
9A	+AAA	10	0.552	12	0.137	14	0.344
9B	+CP	10	0.0365*	12	6.06e-04*	14	0.107
9B	+CP-Ser	10	0.0876	12	0.00251*	14	0.204
9B	+Δ101	10	0.154	12	0.00202*	14	0.102
9B	+AAA	10	0.251	12	0.0192*	14	5.54e-05*
10A	+CP	10	0.0480*	12	0.326	14	0.0258*
10A	+S100A8	10	0.354	12	0.981	14	0.296
10A	+S100A9(C3S)	10	0.205	12	0.141	14	0.0507
10A	+S100A12	10	0.00141*	12	0.654	14	0.813
10B	+CP	10	0.0136*	12	0.00145*	14	0.0148*
10B	+S100A8	10	1.49e-04*	12	0.0516	14	0.255
10B	+S100A9(C3S)	10	0.214	12	0.725	14	0.181
10B	+S100A12	10	0.00409*	12	0.0167*	14	0.249
11A	+CP	10	0.272	12	0.0931	14	0.852
11A	+mCP-Ser	10	0.678	12	0.0626	14	0.0414*
11B	+CP	10	0.0365*	12	6.06e-04*	14	0.107
11B	+mCP-Ser	10	0.0596	12	1.25e-04*	14	6.31e-05*

[a]All significance testing at the endpoint (14 h) was performed with respect to the metal-replete condition using a two-sample unpaired student's t-test. Instances where the p-value met the significance threshold of $p < 0.05$ are marked with an asterisk (*).

SUPPLEMENTARY FIGURES

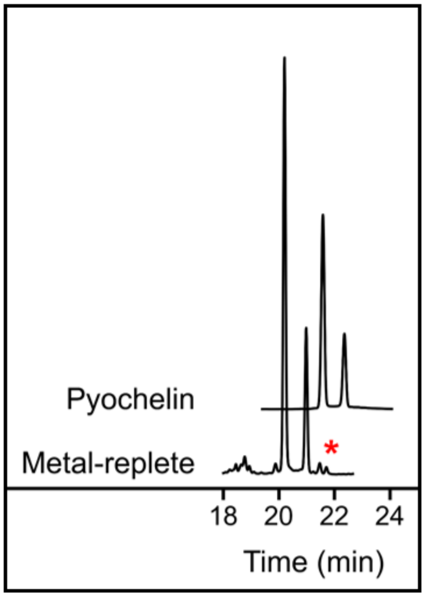

Figure S1. Detection of methylated PCH (21.7 min, red asterisk) in supernatants from cocultures of *P. aeruginosa* and *S. aureus*. A representative HPLC chromatogram is plotted alongside a chromatogram of a PCH standard; PCH elutes as two diastereomeric peaks (20.3 min, 21.0 min) (4, 9). Cultures were grown in metal-replete CDM and incubated at 37 °C for 14 h.

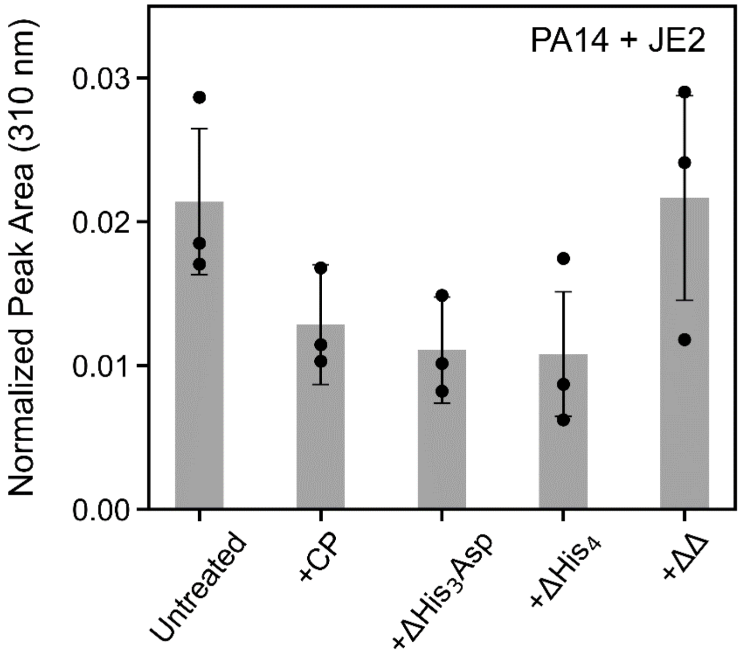

Figure S2. The presence of CP does not significantly affect the relative abundance of methylated PCH detected in cocultures of *P. aeruginosa* and *S. aureus*. Peak area corresponding to methylated PCH was normalized to both the concentration of pyochelin and the log(CFU/mL) of *P. aeruginosa* for each culture. Cultures were grown in metal-replete CDM ±20 μM CP or CP site variant and incubated at 37°C for 14 h (n=3, error bars represent S.E.).

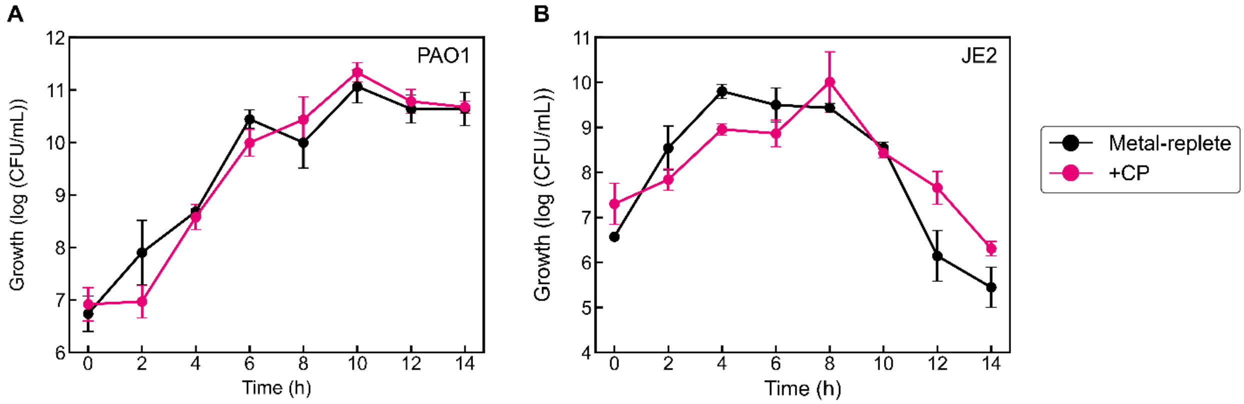

Figure S3. Effect of CP on *P. aeruginosa* PAO1 (A) and *S. aureus* JE2 (B) growth in coculture. Cultures were grown in metal-replete CDM ±20 μM of CP and incubated at 37 °C (n = 3; error bars represent S.E.).

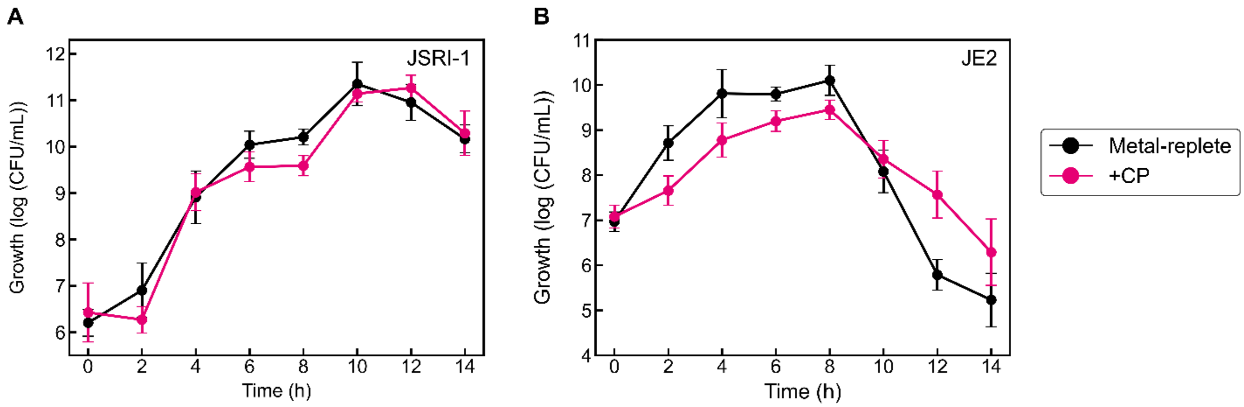

Figure S4. Effect of CP on *P. aeruginosa* JSRI-1 (A) and *S. aureus* JE2 (B) growth in coculture. Cultures were grown in metal-replete CDM ±20 µM of CP and incubated at 37 °C (n = 3; error bars represent S.E.).

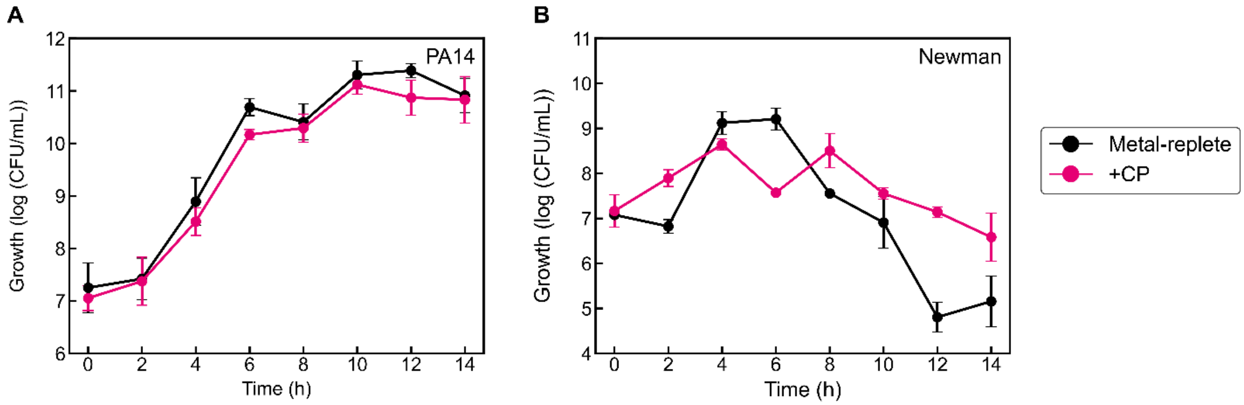

Figure S5. Effect of CP on *P. aeruginosa* PA14 (A) and *S. aureus* Newman (B) growth in coculture. Cultures were grown in metal-replete CDM ±20 µM of CP and incubated at 37 °C (n = 3; error bars represent S.E.).

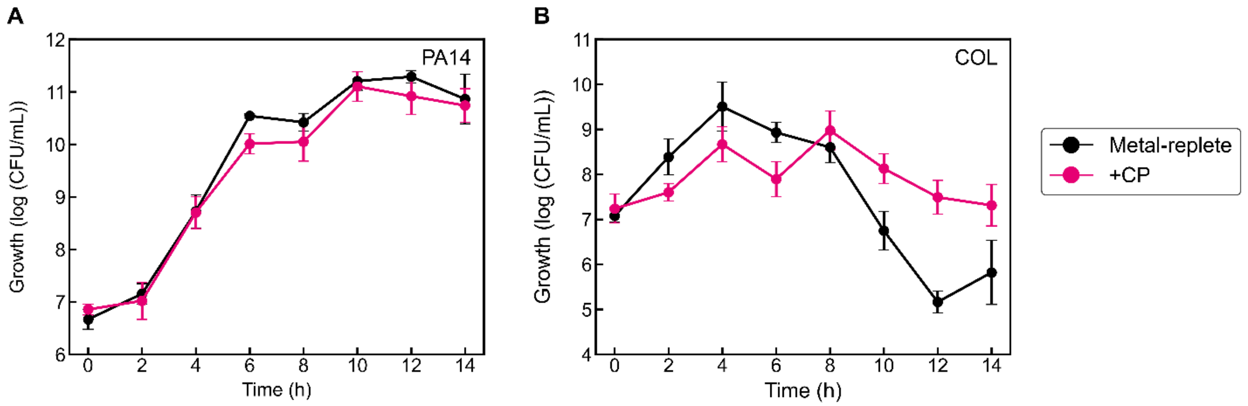

Figure S6. Effect of CP on *P. aeruginosa* PA14 (A) and *S. aureus* COL (B) growth in coculture. Cultures were grown in metal-replete CDM ±20 μM of CP and incubated at 37 °C (n = 3; error bars represent S.E.).

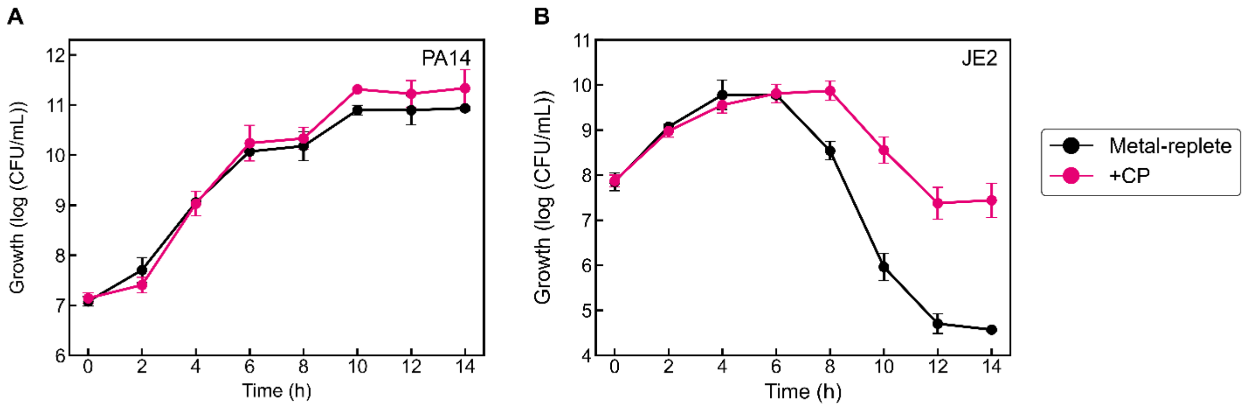

Figure S7. Effect of CP on *P. aeruginosa* PA14 (A) and *S. aureus* JE2 (B) growth in coculture in Tris:TSB medium. Cultures were grown at 37 °C in Tris:TSB medium supplemented with 2 mM Ca(II) and treated with 20 μM of CP (n = 3; error bars represent S.E.).

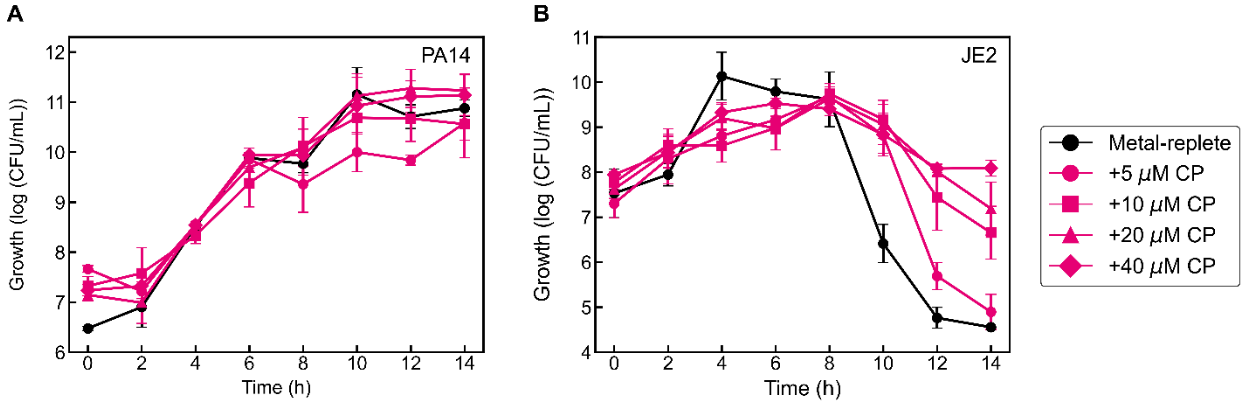

Figure S8. Effect of CP concentration on *P. aeruginosa* PA14 (A) and *S. aureus* JE2 (B) growth in coculture. Cultures were grown in metal-replete CDM, treated with 5 – 40 μM CP where applicable and incubated at 37 °C (n ≥ 3; error bars represent S.E.).

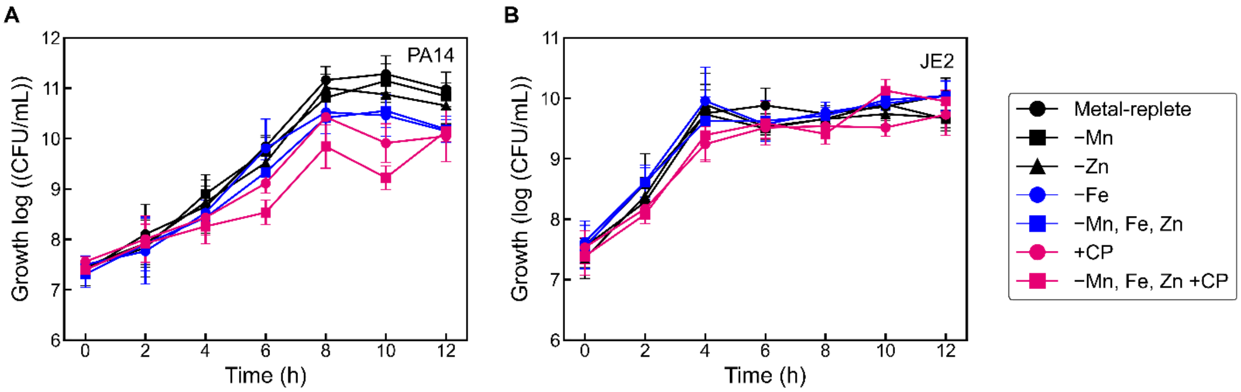

Figure S9. Effect of CP and metal depletion on monocultures of *P. aeruginosa* (A) and *S. aureus* (B). CP treatment and growth in Fe-depleted medium decreases the viability of *P. aeruginosa* monocultures, but does not significantly affect viability of *S. aureus* monocultures. Monocultures were inoculated using a 1:100 dilution from overnight cultures, and grown in metal-replete CDM, single metal-depleted CDM or depleted CDM ± 20 μM CP and incubated at 37°C. (n ≥ 3; error bars represent S.D.).

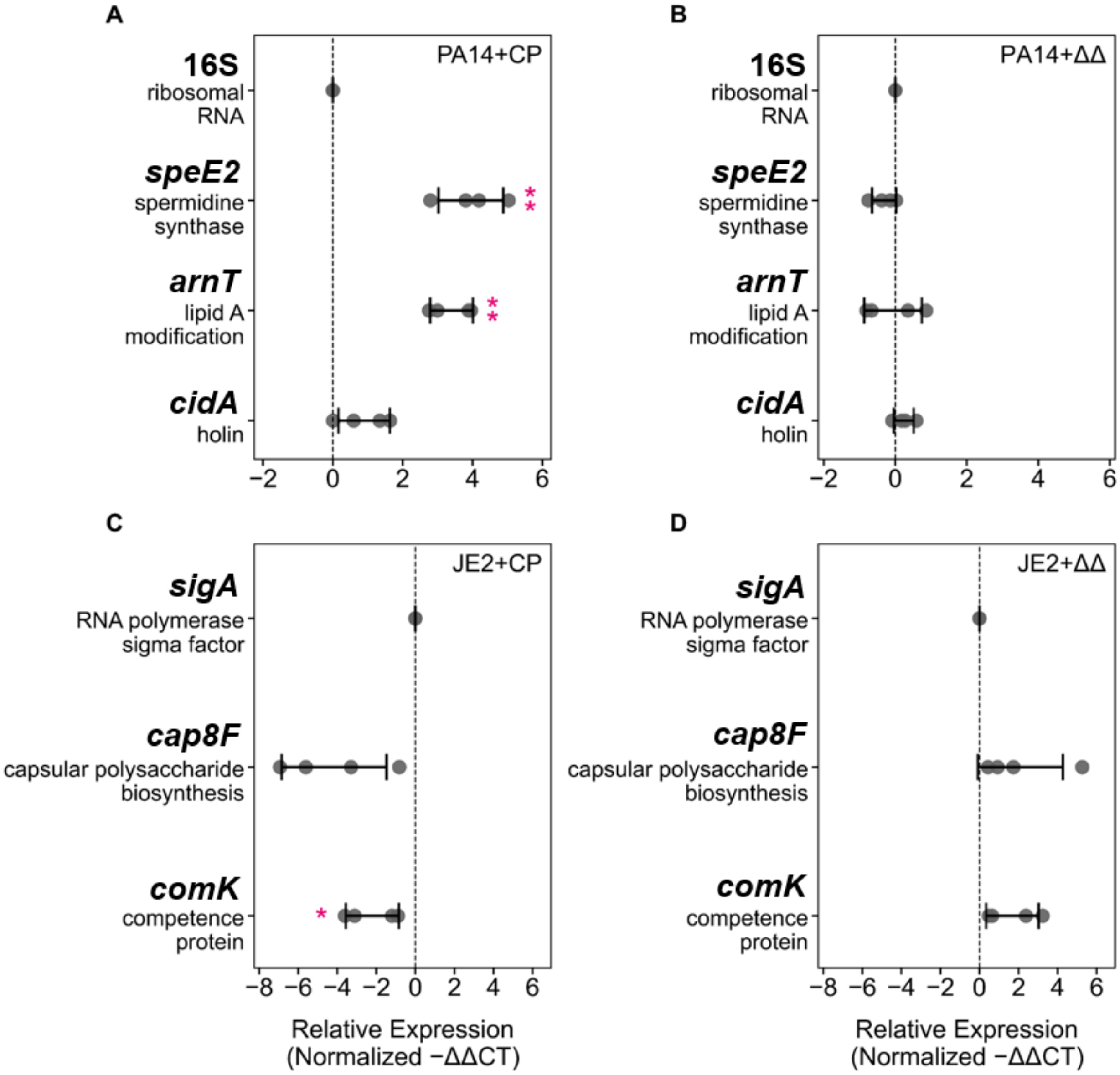

Figure S10. The CP protein scaffold does not perturb the expression of genes that affect the cell envelopes of *P. aeruginosa* (A, B) and *S. aureus* (C, D) in monoculture. Treatment with CP (A) but not ΔΔ (B) induced membrane modifications in *P. aeruginosa* monocultures. While CP (C) slightly downregulated the expression of genes associated with membrane modifications in *S. aureus* monocultures, treatment with ΔΔ (D) instead resulted in a slight increase in expression that was not statistically significant. Transcript levels were normalized to the respective housekeeping genes (*P. aeruginosa*: 16S; *S. aureus*: *sigA*), and the fold change after normalization is presented (n=4, ** $p < 0.01$, * $p < 0.05$, error bars represent S.D.). Cultures were grown in metal-replete CDM ± 20 µM CP and incubated at 37°C for 6 h.

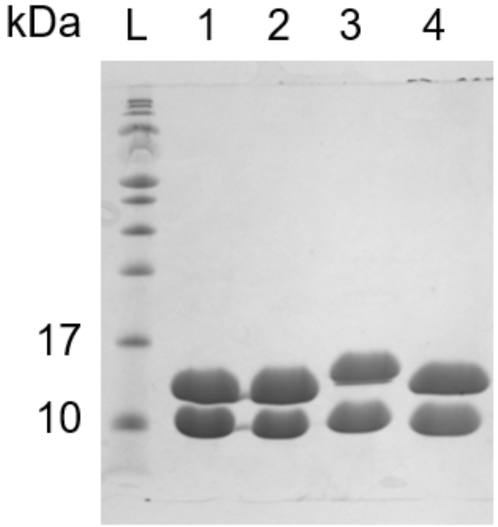

Figure S11. Representative SDS-PAGE (15% Tris-glycine gel) of purified CP and CP site variants utilized in this study. L: P7719S Color Prestained Protein Standard, Broad Range (New England Biolabs). Lane 1: CP; Lane 2: CP ΔHis_3Asp; Lane 3: CP ΔHis_4; Lane 4: CP $\Delta\Delta$.

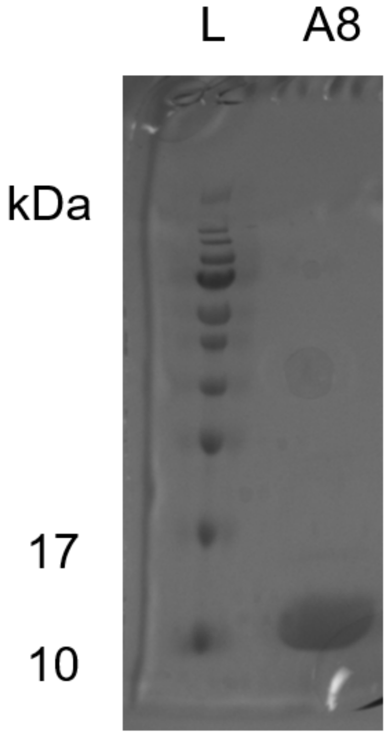

Figure S12. Representative SDS-PAGE (15% Tris-glycine gel) of purified S100A8 utilized in this study. L: P7719S Color Prestained Protein Standard, Broad Range (New England Biolabs).

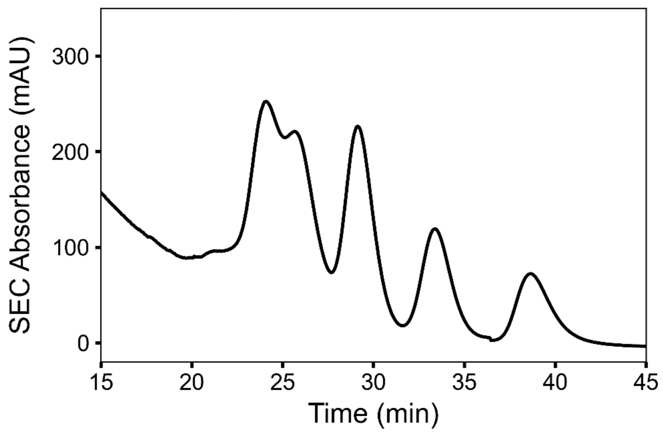

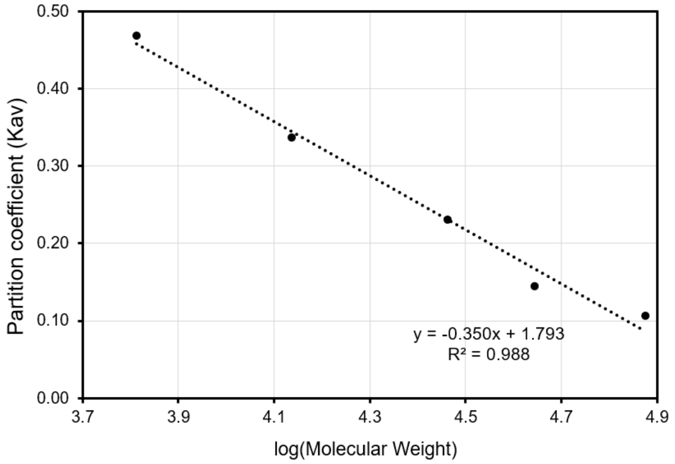

Figure S13. (Top) Analytical SEC trace of LMW standards (Cytiva) used for standard curve determination. Protein peaks (left-to-right): conalbumin (24.1 min), ovalbumin (25.7 min), carbonic anhydrase (29.1 min), ribonuclease A (33.4 min), and aprotinin (38.7 min). The flow rate used was 0.3 mL/min. (Bottom) The standard curve derived from LMW standards (1).

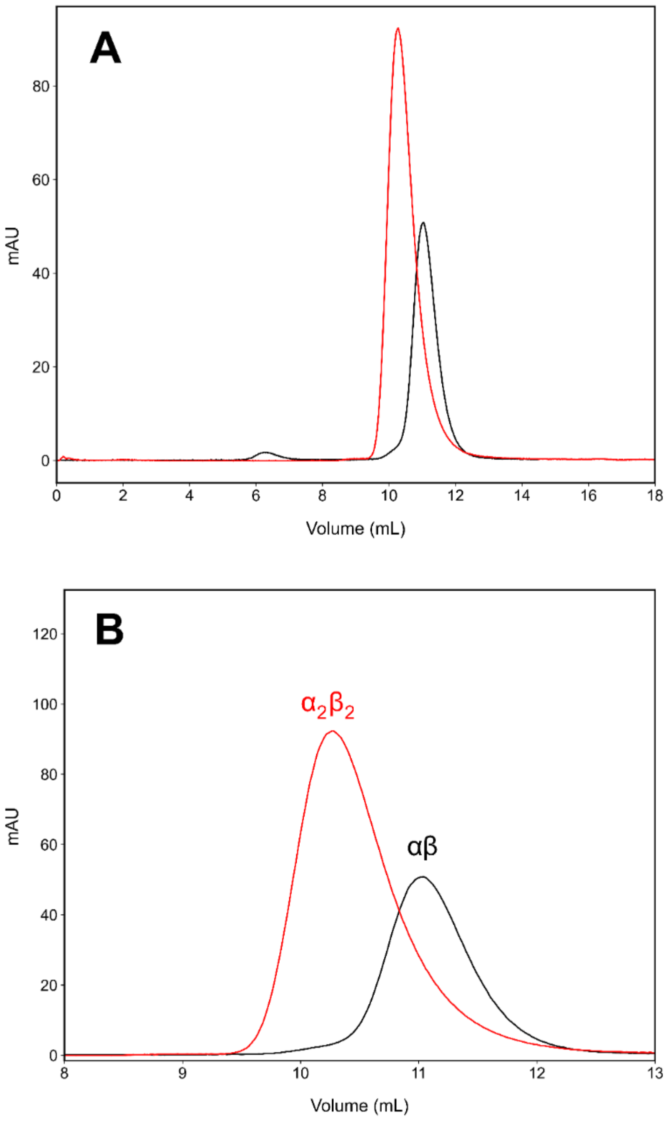

Figure S14. Analytical size exclusion chromatography of 120 µM CP ΔHis$_3$Asp in the absence (black) and presence (red) of 2.4 mM Ca(II) in the running buffer (20 mM HEPES, 150 mM NaCl, pH 8.0). (A) Full chromatograms and (B) an expansion of the main peaks are shown.

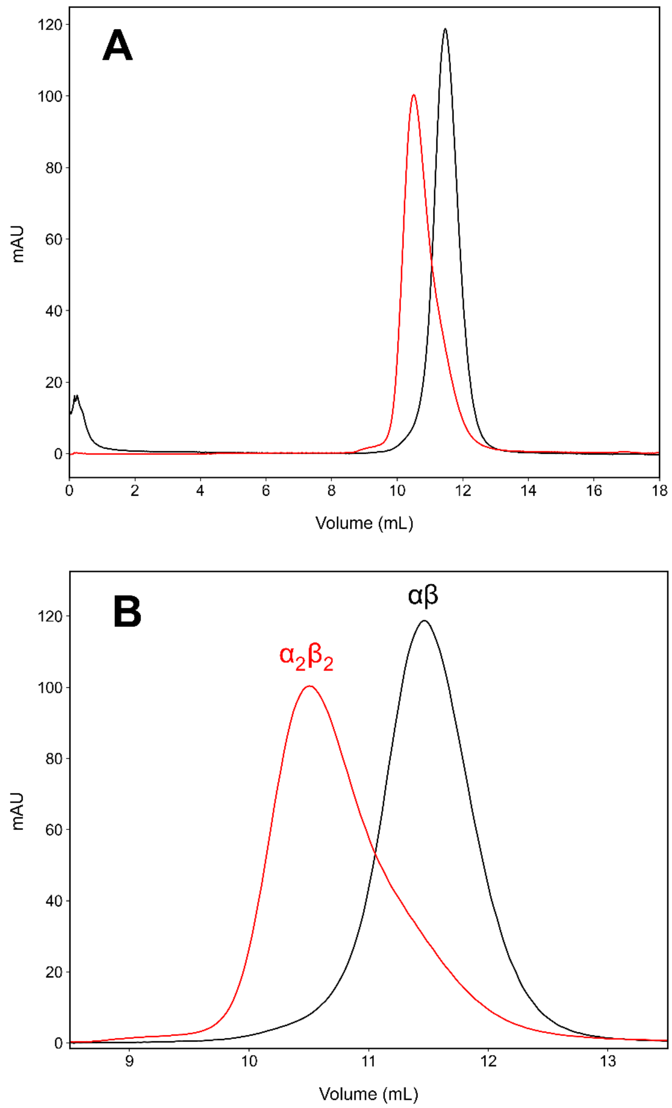

Figure S15. Analytical size exclusion chromatography of 120 μM CP ΔHis_4 in the absence (black) and presence (red) of 2.4 mM Ca(II) in the running buffer (20 mM HEPES, 150 mM NaCl, pH 8.0). (A) Full chromatograms and (B) an expansion of the main peaks are shown.

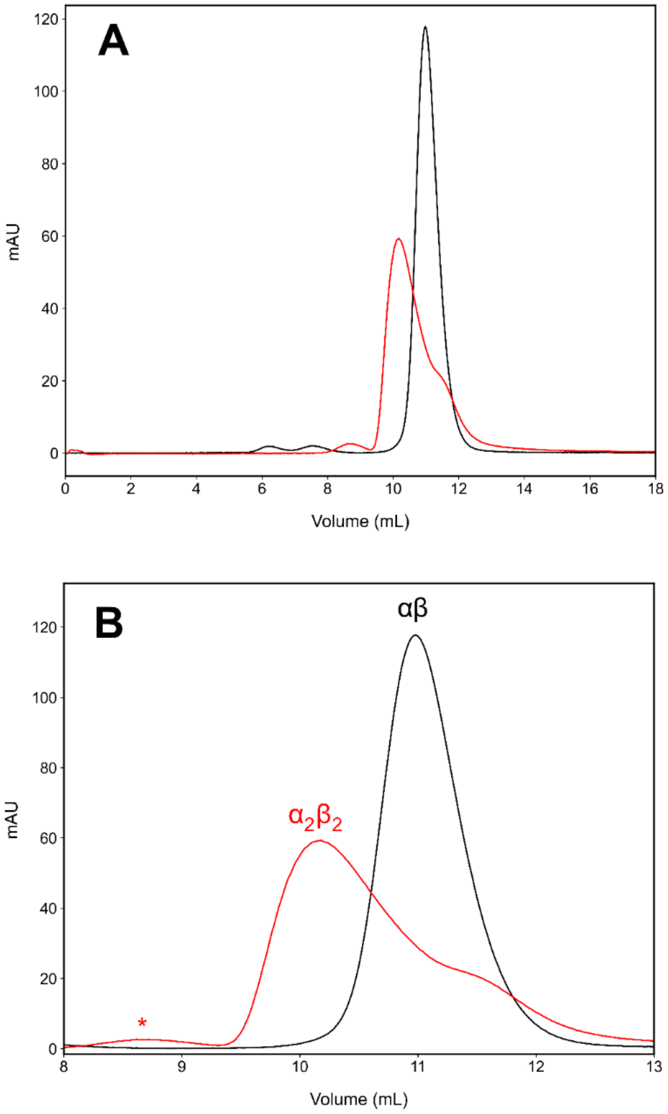

Figure S16. Analytical size exclusion chromatography of 120 μM CP ΔΔ in the absence (black) and presence (red) of 2.4 mM Ca(II) in the running buffer (20 mM HEPES, 150 mM NaCl, pH 8.0). (A) Full chromatograms and (B) an expansion of the main peaks are shown. Peaks that are attributed to Ca(II) higher-order oligomers are marked with red asterisks.

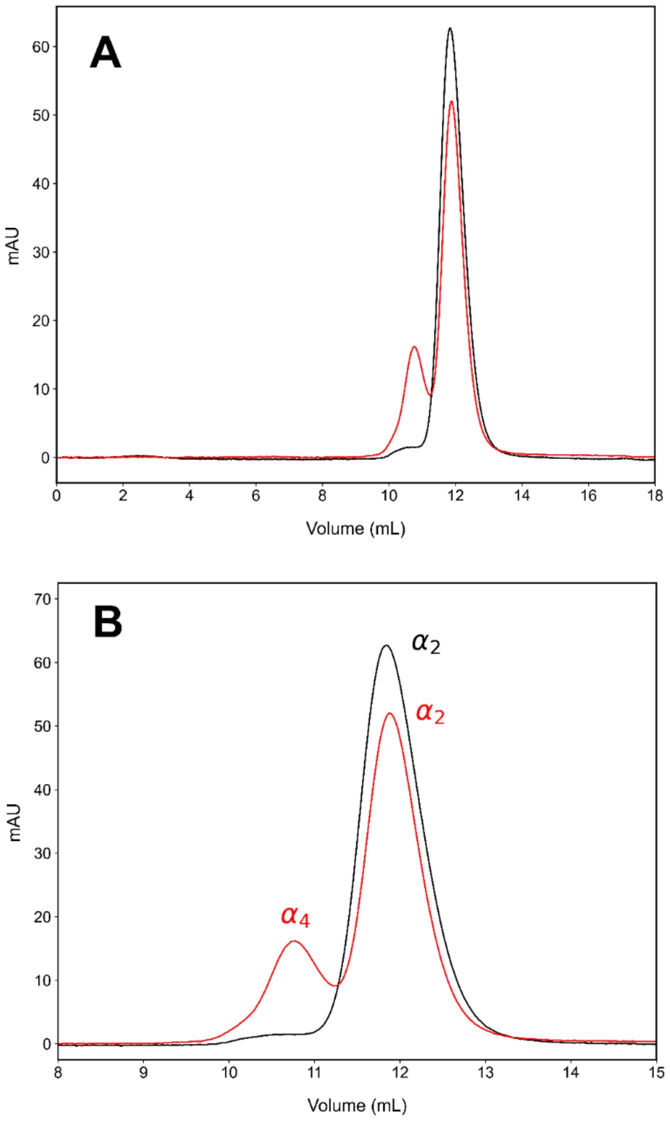

Figure S17. Analytical size exclusion chromatography of 120 μM S100A8 in the absence (black) and presence (red) of 2.4 mM Ca(II) in the running buffer (20 mM HEPES, 150 mM NaCl, pH 8.0). (A) Full chromatograms and (B) an expansion of the main peaks are shown.

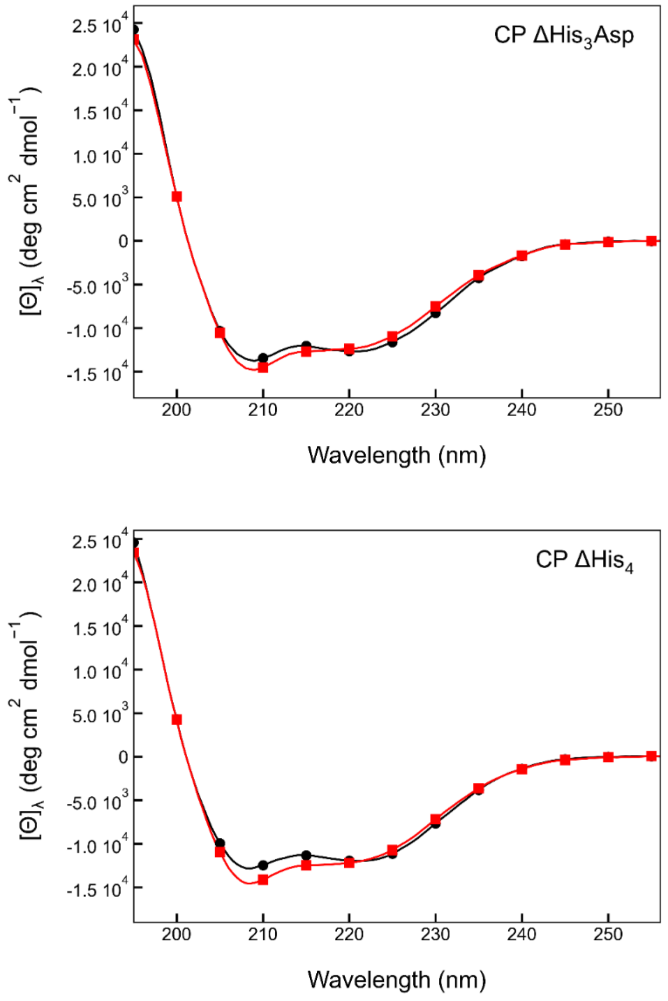

Figure S18. Circular dichroism spectra of 10 μM CP ΔHis$_3$Asp (top) and CP ΔHis$_4$ (bottom) in the absence (black) or presence (red) of 2 mM Ca(II). Samples were collected in 1 mM Tris-HCl, 0.5 mM EDTA pH 8.5 at 25 °C.

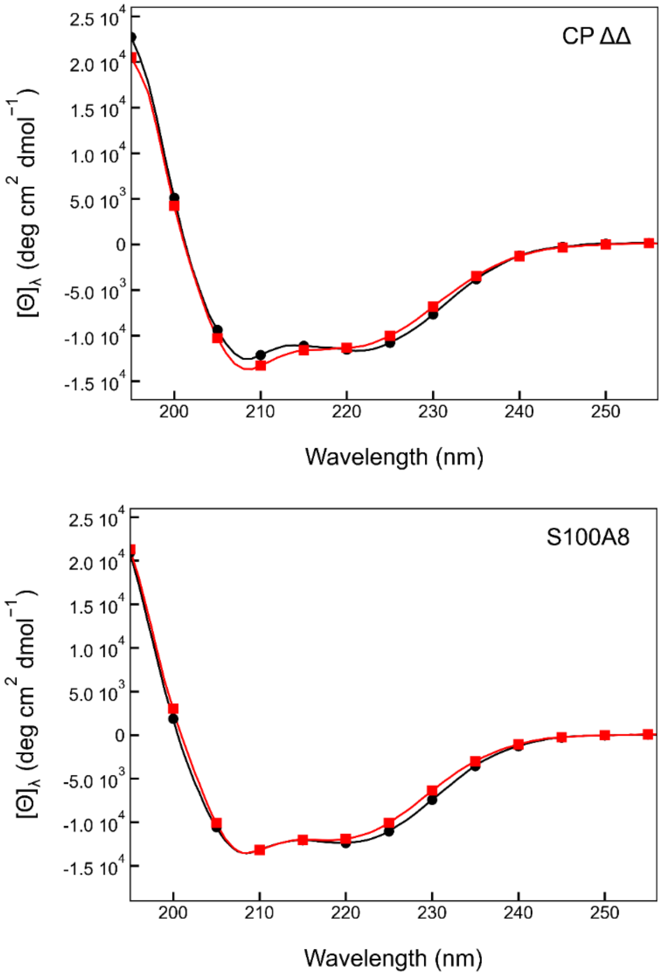

Figure S19. Circular dichroism spectra of 10 μM CP ΔΔ (top) and S100A8 (bottom) in the absence (black) or presence (red) of 2 mM Ca(II). Samples were collected in 1 mM Tris-HCl, 0.5 mM EDTA pH 8.5 at 25 °C.

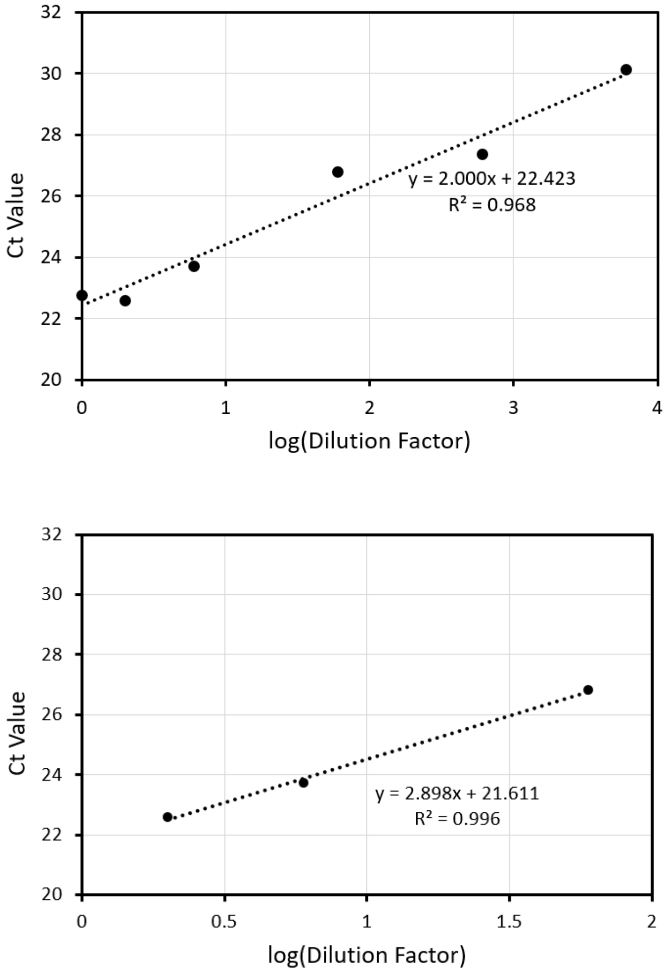

Figure S20. Representative plots used for determination of standard curves for real-time PCR. RNA was extracted from cocultures of *P. aeruginosa* and *S. aureus* grown in metal-replete CDM at 37 °C for 6 h. (Top) C_T values obtained for *sigA* from cDNA synthesis reactions performed using 0.1, 1, 10, 100, 300 and 600 ng of RNA were plotted against the log of dilution factor (taken to be equal to one for 600 ng of RNA, log of which gives zero). (Bottom) To identify the effective dynamic range, problematic datapoints were omitted.

SUPPLEMENTARY REFERENCES

1. Brophy MB, Hayden JA, Nolan EM. 2012. Calcium ion gradients modulate the zinc affinity and antibacterial activity of human calprotectin. J Am Chem Soc 134:18089–18100.

2. Brunjes Brophy M, Nakashige TG, Gaillard A, Nolan EM. 2013. Contributions of the S100A9 C-terminal tail to high-affinity Mn(II) chelation by the host-defense protein human calprotectin. J Am Chem Soc 135:17804–17817.

3. Hadley RC, Gu Y, Nolan EM. 2018. Initial biochemical and functional evaluation of murine calprotectin reveals Ca(II)-dependence and its ability to chelate multiple nutrient transition metal ions. Biochemistry 57:2846–2856.

4. Weiner JM, Lee WH, Nolan EM, Oglesby AG. 2025. Calprotectin elicits an aberrant iron starvation response in *Pseudomonas aeruginosa* under anaerobic conditions. J Bacteriol https://doi.org/10.1128/JB.00029-25R1.

5. Zygiel EM, Nelson CE, Brewer LK, Oglesby-Sherrouse AG, Nolan EM. 2019. The human innate immune protein calprotectin induces iron starvation responses in *Pseudomonas aeruginosa*. J Biol Chem 294:3549–3562.

6. Hunter MJ, Chazin WJ. 1998. High level expression and dimer characterization of the S100 EF-hand proteins, migration inhibitory factor-related proteins 8 and 14. J Biol Chem 273:12427–12435.

7. Nakashige TG, Zygiel EM, Drennan CL, Nolan EM. 2017. Nickel sequestration by the host-defense protein human calprotectin. J Am Chem Soc 139:8828–8836.

8. Atshan SS, Shamsudin MN, Lung LTT, Ling KH, Sekawi Z, Pei CP, Ghaznavi-Rad E. 2012. Improved method for the isolation of RNA from bacteria refractory to disruption, including *S. aureus* producing biofilm. Gene 494:219–224.

9. Adler C, Corbalán NS, Seyedsayamdost MR, Pomares MF, de Cristóbal RE, Clardy J, Kolter R, Vincent PA. 2012. Catecholate siderophores protect bacteria from pyochelin toxicity. PLoS ONE 7:e46754.

10. Cox CD, Graham R. 1979. Isolation of an iron-binding compound from *Pseudomonas aeruginosa*. J Bacteriol 137:357–364.

11. Magalhães AP, França Â, Pereira MO, Cerca N. 2019. RNA-based qPCR as a tool to quantify and to characterize dual-species biofilms. Sci Rep 9:13639.

12. Reinhart AA, Powell DA, Nguyen AT, O'Neill M, Djapgne L, Wilks A, Ernst RK, Oglesby-Sherrouse AG. 2015. The *prrF*-encoded small regulatory RNAs are required for iron homeostasis and virulence of *Pseudomonas aeruginosa*. Infect Immun 83:863–875.

13. Zygiel EM, Obisesan AO, Nelson CE, Oglesby AG, Nolan EM. 2021. Heme protects *Pseudomonas aeruginosa* and *Staphylococcus aureus* from calprotectin-induced iron starvation. J Biol Chem 296:100160.

14. Mozaheb N, Rasouli P, Kaur M, Van Der Smissen P, Larrouy-Maumus G, Mingeot-Leclercq M-P. 2023. A Mildly Acidic Environment Alters *Pseudomonas aeruginosa* Virulence and Causes Remodeling of the Bacterial Surface. Microbiol Spectr 11:e0483222.

15. Holloway BW. 1955. Genetic recombination in *Pseudomonas aeruginosa*. J Gen Microbiol 13:572–581.

16. Rahme LG, Stevens EJ, Wolfort SF, Shao J, Tompkins RG, Ausubel FM. 1995. Common virulence factors for bacterial pathogenicity in plants and animals. Science 268:1899–1902.

17. Nguyen AT, O'Neill MJ, Watts AM, Robson CL, Lamont IL, Wilks A, Oglesby-Sherrouse AG. 2014. Adaptation of iron homeostasis pathways by a *Pseudomonas aeruginosa* pyoverdine mutant in the cystic fibrosis lung. J Bacteriol 196:2265–2276.

18. Fey PD, Endres JL, Yajjala VK, Widhelm TJ, Boissy RJ, Bose JL, Bayles KW. 2013. A genetic resource for rapid and comprehensive phenotype screening of nonessential *Staphylococcus aureus* genes. mBio 4:e00537-12.

19. Baba T, Bae T, Schneewind O, Takeuchi F, Hiramatsu K. 2008. Genome sequence of *Staphylococcus aureus* strain Newman and comparative analysis of staphylococcal genomes: polymorphism and evolution of two major pathogenicity islands. J Bacteriol 190:300–310.

20. Gill SR, Fouts DE, Archer GL, Mongodin EF, DeBoy RT, Ravel J, Paulsen IT, Kolonay JF, Brinkac L, Beanan M, Dodson RJ, Daugherty SC, Madupu R, Angiuoli SV, Durkin AS, Haft DH, Vamathevan J, Khouri H, Utterback T, Lee C, Dimitrov G, Jiang L, Qin H, Weidman J, Tran K, Kang K, Hance IR, Nelson KE, Fraser CM. 2005. Insights on evolution of virulence and resistance from the complete genome analysis of an early methicillin-resistant *Staphylococcus aureus* strain and a biofilm-producing methicillin-resistant *Staphylococcus epidermidis* strain. J Bacteriol 187:2426–2438.
